# Possible roles of neuropeptide/transmitter and autoantibody modulation in emotional problems and aggression

**DOI:** 10.3389/fpsyt.2024.1419574

**Published:** 2024-09-24

**Authors:** Henning Værøy, Regina Skar-Fröding, Elin Hareton, Sergueï O. Fetissov

**Affiliations:** ^1^ R&D Department, Division of Mental Health Services, Akershus University Hospital, Lørenskog, Norway; ^2^ Department of Multidiciplinary Laboratory Medicine and Medical Biochemistry, (TLMB), Akershus University Hospital, Lørenskog, Norway; ^3^ Neuroendocrine, Endocrine and Germinal Differentiation and Communication Laboratory, Inserm UMR1239, University of Rouen Normandie, Rouen, France

**Keywords:** neuropeptides, autoantibodies, emotional problems, aggression, psycho-immunology

## Abstract

The theoretical foundations of understanding psychiatric disorders are undergoing changes. Explaining behaviour and neuroendocrine cell communication leaning towards immunology represents a different approach compared to previous models for understanding complex central nervous system processes. One such approach is the study of immunoglobulins or autoantibodies, and their effect on peptide hormones in the neuro-endocrine system. In the present review, we provide an overview of the literature on neuropeptide/transmitter and autoantibody modulation in psychiatric disorders featuring emotional problems and aggression, including associated illness behaviour. Finally, we discuss the role of psycho-immunology as a growing field in the understanding of psychiatric disorders, and that modulation and regulation by IgG autoAbs represent a relatively new subcategory in psycho-immunology, where studies are currently being conducted.

## Introduction

A field in which psychiatric disorders have an association with the immune system has gradually emerged over the years. Explaining behaviour and neuroendocrine cell communication leaning towards immunology represents a different approach compared to previous models for understanding complex central nervous system (CNS) processes. One such approach is the study of immunoglobulins (Ig) or autoantibodies (autoAbs), and their effect on peptide hormones in the neuro-endocrine system. Research has shown that gut microbes have an impact on the neuroendocrine system (1), and that bacteria are able to respond to stressor-induced neuroendocrine factors of the sympathetic nervous system (SNS) and the HPA-axis, and glucocorticoids can affect bacterial metabolism (2, 3). Further studies have favoured the hypothesis that molecular mimicry is a mechanism by which specific neuropeptides are connected to gut proteins synthesised by (e.g., the Enterobacteriaceae via the immune system; 4). Hence, among the most recent advances in research on behaviour, is the expanded impact of the human microbiota and the modulation by autoantibodies (autoAbs). Microbes in the gut microbiota produce proteins, which enter the bloodstream and by molecular mimicry with the host’s naturally existing neuropeptides stimulate production of autoAbs cross-reactive with these neuropeptides (5–9). These autoAbs, as any circulating IgG, have limited access to the CNS. However, if they pass the blood-brain barrier, they seem to modulate the neuronal activity in the brain regions normally regulating human behaviour (10). Studies have confirmed that immunoglobulin G (IgG) autoAbs have the potential to influence the regulation of at least some parts of human behaviour mediated by adreno-corticotropic hormone (ACTH) (11). In addition, autoAbs may act as carrier proteins of active neurohormones to distant sites, followed by receptor activation (12). The types of activated receptors and different kinetic properties contribute to the explanation of e.g., why some cellular responses are more rapid, whereas others are stronger and longer lasting. However, much work remains to be done before conclusions can be drawn. The first section in this review presents an introduction to the definition of human behaviour, while the following sections cover an update of immunological findings and neuropeptide/transmitter and autoantibody modulation in psychiatric disorders featuring emotional problems and aggressive behaviour.

## Human behaviour

Several attempts have been made to define human behaviour, and in-depth studies have resulted in explanations similar to those provided by Hutchison (13). In brief, human behaviour is the way individuals express themselves mentally, physically, and socially, alone or in groups, as a response to triggers (13). Behind behaviour lie genetic and environmental properties, as well as emotions and mindset. The behaviour displayed by other people when we observe them, results from values and attitudes reflecting individual inherent thoughts and feelings. This makes it possible, to some degree, to decipher and understand someone’s individual psychological traits and personality. However, there are biases linked to the observer’s interpretations of what is seen, causing uncertainty.

In the context of emotional and aggressive disorders, there is associated illness behaviour (IB), a concept often referred to. The difference between symptoms and IB can be difficult to explain, but for the latter it depends on how a symptom is perceived, elaborated and finally acted upon by the patient.

One description of IB states that “The concept of illness behaviour, describes the ways persons respond to bodily indications and the conditions under which they come to view them as abnormal. Illness behaviour thus involves the way persons monitor their bodies, define and interpret their symptoms, take remedial action, and utilise various sources of help as well as the more formal health-care system. It also is concerned with how people monitor and respond to symptoms and symptom change over the course of an illness and how this affects behaviour, remedial actions taken, and response to treatment” (14). A more recent suggestion states that: “Illness behaviour refers to peoples’ experiences and interpretations of the symptoms and illness/disease/injury etc., and their interactions with various social networks as they try to cope with or accommodate these symptoms”, (15).

In brief, suffering from a medical condition will influence your behaviour. Thus, the complexity of human behaviour is immense, and the number of various levels of biological substrates involved is at present beyond the reach of our full understanding. An example of how an illness may have a great impact is COVID-19, which through immune response has caused both morbidity and mortality during and long after the infection (16–18).

## Psychiatric disorders featuring emotional problems

We have learned that anxiety and depression occur quite frequently in our population, and traditionally, many readers associate the term emotional disorders with exactly those two conditions. Extensive research has established that emotional disorders are the most common psychological disturbances, and during our lifespan, these conditions may cause notable impairment (19–21). In addition to their frequent occurrence, many patients wait to seek immediate treatment for the conditions, a factor that potentially exacerbates both personal and economic costs (22).

It has been suggested that the frequent use of the term emotional disorders may lead to an increased risk for confusion since it may reflect different meanings (23). Considering emotional disorders as something beyond anxiety and depression, it has been proposed to incorporate borderline personality disorder (BPD), eating disorders (ED) and insomnia (23). In this paper, we chose to apply this recent definition and expand the topic to psychiatric disorders featuring emotional problems.

### Eating disorders

Disorders such as anorexia and bulimia are the two main eating disorders (ED), both of which are classified as neuropsychiatric disorders due to altered behaviour resulting from brain dysfunction (5, 24). There is increasing support that the underlying etiology of ED seems to be a latent infection with Enterobacteriaceae, causing the production of IgG autoAbs cross-reactive with alpha(α)-melanocyte-stimulating hormone (α-MSH), (5). The paper also provide a more detailed discussion about the postulated role of aMSH-reactive IgG in ED including their antigenic origin in gut microbiota. α-MSH is a neuropeptide of the melanocortin (MC) system inducing satiety via the MC type 4 receptor (MC4R) (25, 26). An Escherichia coli-derived protein, caseinolytic protease B (ClpB), was found to play a role of α-MSH-antigen mimetic (9). In bacteria ClpB functions as a chaperone protein assisting in the unfolding of proteins in the cell following heat shock and other stress (27, 28), thereby preventing random aggregation. There is activation of MC4R receptors by ClpB induced α-MSH cross-reactive IgG autoAbs forming immune complexes (IC) with α-MSH (6, 29). Still, autoAbs, reactive with other regulatory peptides like ghrelin and corticotrophin, could also play a part in the pathophysiology of ED, influencing the regulation of appetite (30) and stress response (11, 25, 26). Moreover, the role of α-MSH-binding IgG as a natural α-MSH-carrier protein in the blood was demonstrated in a recent publication which also revealed the significant correlation of α-MSH-reactive IgG and BMI in both healthy adolescents and patients with anorexia nervosa (31).

### Borderline personality disorder

Borderline personality disorder affects up to 2.7% of the population and is linked to functional impairment and suicide (32). A case linking classic BPD with fluctuating mood and antithyroglobulin antibody titres measured over a period of ¾ of a year suggested a clinically significant, longitudinal correlation between fluctuating antithyroid antibody titres and symptoms of borderline (33). As part of a multi-factorial influence on the development of BPD (34–36), inflammation has been suggested as a possible risk factor (37).

One study, looking at the link between autoimmunity and psychotic symptoms, described an association between DNA hydrolysing IgG catalytic antibodies (DNase activity) and the Positive and Negative Symptoms Scale (PANSS) and Montgomery Aasberg Depression Rating Scale (MADRS) in two subgroups of patients: one with BPD and one with schizophrenia (SCZ). In the two groups of patients studied, the levels of interleukin-6 (IL-6) and total IgG in BPD patients were higher than in SCZ and healthy controls, indicating a relative inflammatory nature of BPD, while autoimmune comorbidity was mainly observed in SCZ patients (38).

Studies have also described decreased levels of brain-derived neurotropic factor (BDNF) in platelets from patients with BPD (39), but this has not been confirmed (40). Other studies have reported a lower level of BDNF and a higher level of tumor necrosis factor (TNF)-α (41) and interleukin (IL)-6 in peripheral blood (42), and there are also reports of increased plasma levels of oxidative stress markers, such as malondialdehyde (MDA) and 8-hydroxy-2-deoxyguanosine (8-OHdG) (43).

A study on inflammatory and oxidative biomarker alterations in borderline personality disorder (BPD) described two clusters of BPD patients. Inflammatory and nitrosative proteins (TBARS, nitrates, catalase, GPx and SOD) were measured in 69 patients with BPD. The results revealed that based on the results, the patients could be clustered in 2 subgroups where in one there was increased anti-inflammatory and increased antioxidant levels and longer disease chronicity and less acute symptoms such as anxiety (44). An exploratory study on the pathophysiology of BPD with focus on the levels of inflammatory cytokines, brain-derived neurotrophic factor (BDNF) and oxidative stress substances known to enhance neuronal damage, showed that patients with BPD have a lower level of BDNF and a higher level of tumor necrosis factor (TNF)-a and interleukin (IL)-6 in peripheral blood. This was associated with elevated plasma levels of oxidative stress markers, e.g. malondialdehyde and 8-hydroxy-2-deoxyguanosine (32). Another study proposed a possible link between emotional dysregulation and psychopathological similarities between BPD and ED. In addition, trauma exposure was found to be of importance in both disorders. There were no shared inflammatory findings, however it was concluded that several risk factors were present, e.g. eating disorders, trauma and impulsivity as a personality feature, all associated with inflammatory features (45).

### Narcolepsy

Narcolepsy type 1 (NT1) is a chronic sleep disorder where inflammation is proposed as the underlying neurodegenerative mechanism (46–49). NT1 shows a specific adaptive immune response directed towards hypocretin/orexin neurons, thus supporting an autoimmune hypothesis (48, 50). Main features in narcolepsy are sleepiness during daytime and cataplexy due to loss of immunoreactive hypocretin or orexin (ORX) neurons in the hypothalamus (51). Narcolepsy displays emotionally triggered episodes with loss of muscle tone (cataplexy), nocturnal sleep disturbances, sleep paralysis and hypnagogic and hypnopompic hallucinations (46). NT1 is associated with changes in cytokine levels and recently the gut microbiota seems to be involved in the inflammatory development (46, 52), and possible impact from the COVID-19 pandemic (53). Studies have also shown microbial translocation through the gut barrier in narcolepsy patients (54).

Production of T helper 1 lymphocytes (TH1) cytokines regulate a major part of the immune based physiological activity (47), whereas the T-helper 2 cells (TH2) produce anti-inflammatory cytokines which counteract the TH1 response (55). Autoantibodies have been detected in plasma from patients (48), and influenza A and beta infections can cause the onset of narcolepsy (49). Through molecular mimicry, foreign antigens may activate autoreactive T cells or B cells due to similar structure between foreign and self-antigens resulting in autoimmunity (56), supporting that in NT1 patients a T-cell mediated autoimmune origin of NT1 (56, 57). In particular narcolepsy-cataplexy shows higher serum levels of autoantibodies against orexin bound as immune complexes, indicating a possible role in the regulation of the sleep-wake cycle (58).

### Anxiety disorders

Anxiety may be difficult to define, but according to DSM 5 (59) the anxiety disorders include Generalised Anxiety disorder (GAD), Obsessive - Compulsive disorder (OCD), Panic disorder, Post Traumatic Stress disorder (PTSD) and Social phobia. However, a later reclassification has defined PTSD as a trauma/stressor-related disorder. Studies have shown that inflammation can influence signalling in the HPA axis (60) and brain regions of importance for anxiety and fear as seen in PTSD (61). Other studies in support of an association between inflammation and anxiety and depression has also been established (62, 63).

There is a link between immunity and anxiety (64), including a high prevalence of anxiety disorders in patients with immune linked disorders. A very recent study on general anxiety disorder (GAD) reported a few causal links between some immunophenotypes and GAD (65). However, another study (66) explored the previously described association between anxiety and Systemic Lupus Erythematosus (SLE) (67, 68), performing a bidirectional Mendelian randomisation (MR) analysis, and found no support for a causal relationship between SLE and anxiety disorder (66). Neuroinflammation seems to play a role in anxiety (69), involving the corticotropin releasing hormone receptor 2 (CRHR2) in the etiology of the disorder (70, 71).

It is widely accepted that the CNS is targeted by the immune system, but how autoAbs pass through the blood–brain barrier (BBB) is still unclear. Under normal conditions, immunoglobulins go through the BBB at a very low rate; a good example is immunoglobulin G (IgG). IgG concentration in the cerebrospinal fluid (CSF) is approximately 1% of the levels in the peripheral circulation indicating that once the autoantibodies reach the CNS they can cause disease as it has been observed in autoimmune encephalitis, an immune based condition causing non-infectious brain inflammation.

After thorough analysis looking for possible biomarkers, studies have shown that elevated thyroid-stimulating hormone (TSH) and anti-thyroid globulin (TGAb) could function as significant predictors of anxiety in depressed patients (72). Regression analysis has shown that in patients with combined anxiety and depression, both TSH levels and TGAb levels are found to be associated with anxiety. However, the clinical characteristics and factors associated with anxiety varied with the age of onset (73).

### Depression

An association between social stress, depression, cytokines, chronic low-grade inflammation and autoimmune disorders has been shown (74–82). Greater concentrations of C-reactive protein (CRP), interleukin-6 (IL-6) and tumor necrosis factor-α (TNF-α) have also been reported, contributing to the development of the hypothesis focusing on a cytokine/inflammatory pathway in the pathophysiology of depression. Antibodies have also been implicated in the pathophysiology of depression (83) and the role of immune-related mechanisms in depression has been supported by the consistent finding of raised peripheral concentrations of proinflammatory cytokines in the blood of patients with depression (83, 84). However, the relevance of inflammation in depression seems related to coexisting physiological disturbances seen in depressive patients, such as aberrant HPA axis activity and in particular intracellular mitochondrial processes (85, 86). Synaptic plasticity and antibodies (anti-ribosomal-P and anti-N-methyl-D-aspartate receptor antibodies) are deeply related to the pathogenesis of neurodevelopmental disorders, especially depression (83). In addition, elevated TSH, anti-thyroglobulin (TgAb), and thyroid peroxidase antibody (TPOAb) levels have all been linked to depression (87). There are also studies strongly linking depression with concomitant Graves’ disease (GD) suggesting GD as a high risk for depression, although more data is needed (88). Recently, a study showed an increased prevalence of thyroid disease among US adults with depression from 2007 to 2018, with the highest rate found in white non-Hispanic old women (89).

Depressive disorders are also associated with autoAbs against neuropeptide Y and oxytocin an vasopressin (90, 91). Animal studies have also shown depressive behaviour in mice after autoAbs injection and improvement following immunosuppressive treatment, thus AutoAbs and depressive symptoms seem linked in depressive subjects; autoAbs also causing tissue damage to the brain (92).

Pursuing the idea of a cytokine/inflammatory pathway, it is also suggested that chronic, low-grade inflammation plays a part in the maintenance of depression (93, 94). Still, low-grade and subclinical inflammation is not a main feature in all depressed patients, but rather in a subgroup (95). In this regard, studies of neuronal surface antigens (NSAbs) (which are the target of autoAbs) against various antigens have also been suggested to be involved in the pathology of both depression and anxiety, but again, indications are that this could be valid only for small groups of patients (96).

#### The gut microbiota and depression

The gut is the largest endocrine organ in the body (97, 98), and contains a number of intrinsic and interneuron connections in the body’s autonomous nervous system (99, 100). The gut complex seems to be involved in emotional and sensory processing via the gut-brain axis, (101), and studies have shown that exposure to stress may have influence on the production of peptides with antimicrobial effects (102), and furthermore that stress over some time may cause both depression and anxiety. Of interest is that the latter conditions also have been associated with alterations of the composition of the gut microbiota (103–106).

The gut microbiota is composed of several types of commensal microorganisms, including bacteria, yeast, and viruses—microbes that help to maintain the integrity of the mucosal barrier. Communication with a bidirectional dialogue exists between the gut microbiota and the brain and the metabolic activity of the gut microbiota and bioactive metabolites are crucial for a normal brain function and have great influence on the course of many neuropsychiatric disorders (107).

In a study of gut microbiota and depression, the composition of the faecal microbiota found in depressive patients had an overrepresentation of enterobacteria and alistipes. Among the results, there was a negative correlation between Faecalibacterium and depressive symptoms (104). Another study confirmed the differences seen in the gut microbiota composition, showing that in depressive-like rats, a significantly different composition was seen compared to the control animals (108). Others studied patients with Irritable Bowel Syndrome (IBS) coexisting with depression, and found that altered microbial and metabolomic profiles were associated with clinical and psychological symptoms (109), e.g., bacterial phylotypes correlate with anxiety-like behaviour (110). To complicate matters, in a meta-study of mental disorders and gut microbiota composition of 24 patients with major depressive disorder (MDD), 7 with bipolar disorder (BD) and 15 with schizophrenia, there was no convincing evidence for a difference in the number or distribution (α-diversity) of bacteria in those with a mental disorder compared to controls (111). In a more recent study (112), it was described that a high abundance of Candidatus soleaferrea reduced the risk of prenatal depression and that the higher the concentration of propanoic acid, the higher the risk of prenatal depression (112).

The term metabolome refers to the system of qualitative and quantitative collection of low-molecular-weight molecules in a cell, including a various range of metabolites in a biological sample. In brief, the metabolome contains both molecules derived from host endogenous processes and those derived from the microbiota (113). There are recent results emphasising that the gut microbiota responds to changes in female sex hormone status, a finding with potential negative consequences for normal metabolic function (114). The organisms in the microbiota may also provide health benefits to the host (115) and the use of probiotics or faecal microbiota transplants from healthy individuals are examples of what may ease the illness and induce fewer symptoms in patients with psychiatric disorders (115, 116). A study of neonates in a neonatal intensive care unit found that higher levels of neonatal infant stressors were associated with differences in the microbiome compared to infants with lower exposure to neonatal infant stressors (117). Interestingly, these microbiome differences were evident in adulthood even though the adversity occurred in childhood (118), demonstrating that early life stress can have lasting effects on microbiome composition.

Future research on the relationship between the gut microbiota and associated disorders with an imbalance in the microbiota composition is needed (119–121).

#### The gut microbiota and molecular mimicry

The enteric mucosal, endocrine and immune systems, operate intimately with each other, and the three systems share characteristics between entero-endocrine cells, pituitary corticotropic cells and pancreatic islet cells (106), and likewise the possibility for the secretion of polypeptide hormones is shared. For each of these three cellular systems, there are molecules thought of as being unique for each system—cytokines (immune system), neurotransmitters (nervous system), and hormones (endocrine system), which also share a common developmental origin (122). Gut epithelial sensor cells or endocrine cells seem to play a role for the synaptic connection of the intestinal lumen to the brainstem (123), thereby allowing regulation of the gut function and communication with the CNS by secreting hormones, which in turn, may activate local sensory nerves or by establishing synaptic communication with enteric glia structures (124).

A person’s gut microbiota contains high microbial variation and therefore provides continuous antigenic stimulation, maintaining physiological immune activity. Molecular mimicry seems crucial for how microbial proteins in the gut microbiota and various neuropeptides are involved in the regulation of motivated behaviour and emotion. Immunoglobulins reactive with these neuropeptides are present in humans and are associated with neuropsychiatric conditions including depression, anxiety, eating disorders, and sleep conditions (7).

A recent model for understanding the interaction between the gut microbiota and bacterial mimicry is based on the finding that an anorexigenic bacterial protein Escherichia coli caseinolytic protease B was recently found to be responsible for the production of α-MSH-cross-reactive autoantibodies and this protein was also detected in human plasma. The model states that eating disorders such as anorexia nervosa (AN) and bullemia nervosa (BN) may be the result of altered signalling between the gut microbiota and host neuroendocrine and immune systems regulating feeding behaviour. The model includes a specific bacterial antigen mimetic of α-MSH and the triggering of the production of α-MSH cross-reactive autoantibodies (autoAbs). These antibodies form immune complexes (IC) between IgG autoAbs and α-MSH, activating the melanocortin (MC) system; an activation being of great importance for the regulation of feeding behaviour. Exposure to highly immunogenic bacteria such as Salmonella, later in life, as well as medication or environmental-induced intestinal infections and dysbiosis, may lead to increased prevalence of pathogenic Enterobacteriaceae and in turn the production of α-MSH-cross reactive autoAbs in vulnerable persons (5).

#### Anti-Ro52 risk factors

Risk factors for depression have often been reported. Among the best known are a family history of depression, early life abuse and neglect, female sex, and medical illness, especially metabolic and autoimmune disorders. The anti-Ro or anti-Sjogren’s syndrome- related antigen A autoantibodies (SSA antibodies) are among the most frequently detected IgG autoantibodies and is associated with systemic lupus erythematosus (SLE), Sjögren’s syndrome (SjS), subacute cutaneous lupus, and neonatal lupus syndrome (125). A study from 2023 showed that the anti-Ro52 antibody is a risk factor for depression and anxiety in SLE, SjS, rheumatoid arthritis (RA) and other connective tissue diseases (CTDs) (72). Thus, patients with depression have various immune abnormalities, and in CTDs, the risk of mental disorders such as depression and anxiety is increased.

Ro52 is a 52-kDa protein that contains a Real Interesting New Gene (RING) finger domain, B-box motifs and a coiled-coil domain; a protein containing 40-60 amino acids, mediating enzymatic interaction between regulatory proteins. This structural feature places Ro52 within the tripartite motif proteins (TRIM) family and is designated as TRIM21 protein, the latter an intracellular antibody effector, binding among others to IgG. The TRIM proteins family are involved in cellular growth, differentiation, apoptosis and genetic transcription. Ro52 mediates ubiquitination, a specific adeno-three phosphate (ATP) dependent biological process influencing interferon regulatory factors (IRF) through its E3 ubiquitin ligase enzymatic activity, specifically inhibiting the excessive production of type 1 interferons and the subsequent prolonged immune system activation, and in turn, the development of autoimmune diseases (126).

## Aggressive behaviour

Aggression is sometimes considered as a symptom in a co-occurring disorder, often mental; sometimes a behavioural description is added. There seems to be an agreement that aggressive behaviour involves harming people, including self-harm, animals, and/or property. Examples of behaviour can include agitation, hyperarousal, paranoid mindset, mood alterations, quarrelling, delusions, poor judgement, coping problems, disorganised thinking and communication, and depression, to mention some. Thus, the range of symptoms in aggressive behavioural disorders is wide. How to classify aggression is therefore an ongoing debate since new studies bring new results, which are more or less important for the classification of the condition. Traditionally, aggression is divided into two main categories: impulsive and premeditated (127) or instrumental. Impulsive behaviour is recognised by its immediate reaction to provocation and the individual’s loss of behavioural control, whereas premeditated behaviour with few exceptions is not associated with agitation. There is also a response to, e.g., fear, threats, and aggression shown during hunting. This behaviour requires a distinction between affective defence and predatory attack, referring to goal-directed attack (128). Whereas defensive aggression might be considered “normal” behaviour, premeditated and impulsive aggression are pathological. Intermittent explosive disorder (IED) is an aggressive behaviour disorder with specific criteria in which there are changes in the salivary cortisol levels at awakening compared to controls (129–131). IED has lifetime and 12-month prevalences of 7.3% and 3.9%, respectively. In addition, IED has an early age of onset and is associated with comorbid mental disorders that, after some delay, seem to have an older age of (132). Furthermore, results suggest the involvement of two or more gene risk alleles in adolescent violent/aggressive behaviour including impulsivity/impulsive behaviours such as IED and other aggressive and violent behaviours (133).

An aggressive condition featuring wild and uncontrollable anger, is rage. Rage is associated with primary brain structures, including the hypothalamus and midbrain periaqueductal grey (PAG), whereas limbic structures, including the amygdala, hippocampus, septal area, prefrontal cortex, and anterior cingulate gyrus, serve important modulating functions (134, 135). Excitatory neurotransmitters that potentiate rage (136) include excitatory amino acids, substance P, catecholamines, cholecystokinin, vasopressin, and serotonin that acts through 5-HT2 receptors (137). Inhibitory neurotransmitters include GABA, enkephalins, and serotonin that acts through 5-HT1 receptors (137).

In an animal study in 1980, it was concluded that tryptophan-induced increases in brain and spinal cord serotonin content enhance behaviours that depend on serotonin release (138). This was the beginning of what later became known as the serotonin syndrome; a potentially lethal condition, due to the use of serotonergic drugs on peripheral and central postsynaptic 5HT-1A, and specifically 5HT-2A receptors (139). A study on a group of severely aggressive subjects, institutionalised since childhood for mental retardation, as compared with suicide attempters and healthy controls, supported an hypothesis of an abnormal function of the 5HT system in aggressive behaviour (140). However, despite the strongly established inverse relation between serotonin and human aggression, a meta-analysis questioned the established, pointing at the need to revise the serotonin deficiency hypothesis in view of serotonin’s functional complexity (141).

Studies on psycho-immunology have also had an impact on the understanding of aggressive behaviour. In a feline defensive rage model, interleukin-1 beta (IL-1β) has an aggression-facilitating effect when injected directly in the PAG and the medial hypothalamus, and evidence supports a role for brain-derived IL-1β in contributing to individual differences in aggression (142).

Cross-species studies have shown that aggressive encounters increase peripheral cytokine releases in both aggressive and non-aggressive animals and that injection of lipopolysaccharides (LPS) or IL-1β induces sickness behaviour and reduces aggressive behaviour (142). In animal studies where rats were administered LPS, it was shown that the levels of the anti-inflammatory cytokine IL-10, seen in all brain structures of aggressive rats, were decreased (143).

It has been demonstrated that brain cytokines, including IL-1β and interleukin 2 (IL-2), powerfully modulate rage behaviour. IL-1β exerts its actions by acting through 5-hydroxytryptamine 2 (5-HT2) receptors, while IL-2 acts through gamma-aminobutyric acid A (GABAA) or neurokinin 1 (NK1) receptors (144, 145). There is evidence supporting cytokine dysregulation as a possible neuroimmune mechanism underlying aggressive behaviour, a finding of importance for new approaches to the treatment of affective disorders (146). One could perhaps anticipate that some of the ongoing processes would be reflected in the CSF; however, new studies show that following direct application of proinflammatory proteins in the brains of animals, aggressive behaviour is increased, but the influence of the proinflammatory proteins on the behaviour is not reflected in the lumbar CSF (147). It has been shown that prenatal glucocorticoid exposure may influence how fast the stress response is terminated, and that such exposure seems linked to higher levels of a later manifested baseline corticosterone concentrations and the associated acute stress response, thereby suggesting dysregulated negative feedback (148).

Increasing evidence also suggests a role of inflammation and immunologic processes in modulating aggressive behaviour induced following chronic exposure to psychological stress (149), such as cruelty during upbringing. In this regard, it was recently shown that sympathetic nervous system activity may moderate the effects of harsh parenting on later aggression (150).

Of interest is also a study, which found that low narrative coherence and high offender hostility assessment (151, 152), combined with respiratory sinus arrhythmia (RSA) activation, were linked to reactive physical aggression in men but not in women (153). The study also showed that the offender’s hostility was associated with reactive relational aggression for both men and women. Thus, there are indications of gender differences and subtypes of aggressive behavioural responses (153).

One of the existing hypotheses on the understanding of human aggression is based on hormonal models where ACTH IgG autoAbs are known to influence, or more precisely, modulate behaviour (154, 155). Simplified, in the working hypothesis, molecular mimicry is essential, and bacteria and viruses in the human microbiota generate neuropeptides cross-reacting with endogenous substances, giving rise to autoAbs, which in turn have the capability to regulate and modulate behaviour. A study found that thyroid peroxidase (TPO)-Abs can affect aggressive behaviour but do not affect suicidal behaviours in patients with major mental disorders (156). Thus, antibody testing can become important for psychiatric patients with aggressive behaviour. The TPO-Ab test has a history of helping the psychiatric staff to determine aggressive behaviour in advance (156).

### Intermittent explosive disorder

IED has lifetime and 12-month prevalences of 7.3% and 3.9%, respectively (132). Studies have also shown that IED has an early age of onset and is associated with comorbid mental disorders that, after some delay, seem to have an older age of debut (132). Furthermore, results suggest the involvement of two or more gene risk alleles in adolescent violent/aggressive behaviour including impulsivity/impulsive behaviours such as IED and other aggressive and violent behaviours (133). A case-control study found that both plasma C-reactive protein (CRP) and interleukin 6 levels were significantly higher in participants with intermittent explosive disorder compared with psychiatric or normal controls. These inflammatory markers were directly correlated with a composite measure of aggression, and with history of actual aggressive behaviour in all participants (157).

### Antisocial personality disorder

The lifetime prevalence of Antisocial personality disorder (ASPD) in the general population range from approximately 1-4% (158, 159). ASPD is characterised by high levels of impulsivity, psychopathic traits, and a high prevalence of co-morbid substance use disorders (SUDs). Aggression is a frequent manifestation in ASPD and may determine long and recurrent imprisonment (160). Few studies have been made on the relationship between ASPD and the immune system. However, one study found that significantly higher levels of tumor necrosis factor (TNF)-α, lower levels of transforming growth factor (TGF)-β1 and brain-derived neurotrophic factor (BDNF) among patients with ASPD, including for those with ASPD+SUD and SUD alone (161) .

### ACTH and aggression

Regardless of whether it is pathological or not, coping with stress can result in aggressive behaviour (162–164). The hypothalamus, and especially activity in the hypothalamus-pituitary-adrenal axis (HPA) is dominant in anxiety and stress reactions and regulates the coordination and fine interaction between the brain and adrenal cortisol production via ACTH secretion from the hypophysis (165, 166). Both high and low HPA activity are linked to aggressive behaviour (167) and cortisol inhibits HPA activity at all levels, modulating anxious behaviour and lowering testosterone production (168). A study on long-time imprisoned males showed that, compared to controls, naturally existing adrenocorticotropic hormone (ACTH) immunoglobulin G autoAbs (ACTH IgG autoAbs) had specific epitope binding profiles in their binding to the ACTH peptide in a population of male violent criminals. The controls were healthy, non-convicted males recruited from various work sites in normal society. While IgG from non-violent controls was bound to the central part of the ACTH (amino acids 11-24), IgG from the violent aggressors showed a higher affinity for the ACTH N-terminal part (amino acids 1-13). Applying the Resident Intruder Test (RIT) on laboratory rats after intraperitoneal injection of ACTH and IgG from the violent criminals but not from the controls, a shorter latency was observed before the resident’s first attack against the intruder (11).

Taken together, the results showed that ACTH IgG autoAbs in plasma bind different epitopes in violent criminals and healthy controls. Furthermore, the ACTH autoAbs modulate ACTH-induced adrenal cortisol secretion involved in regulation of the stress response (11). Another study on antisocial behaviour, stress, and cortisol response confirms the relationship between the level of ACTH IgG autoAbs, antisocial behaviour, and HPA activity following stress in young people (169. Another study showed that both ACTH, oxytocin, and vasopressin autoAbs are altered in subjects with conduct disorders, with the levels of ACTH-reactive IgG most-affected (170).

### Oxytocin and stress-related behaviour

Oxytocin is an amino acid peptide in the hypothalamus that activates the oxytocin receptor (OTR), triggers the release of Ca2+, and causes receptor internalisation (endocytosis). OT has several functions throughout the body, most of them mapped, e.g., in the central nervous system (CNS) influencing social behaviour (171). *In vivo* real-time recordings of the responses in OT neurons in the hypothalamic paraventricular nucleus (PVN) in mice, recently showed that OT neurons were significantly more activated by stressors than by social stimuli, a finding which correlated with depressive-like behaviour during stress. In addition, inhibition of OT-neurons in the PVN influenced stress-induced social memory by impairing the response, and opening for a role of PVN OT neurons in stress-induced social amnesia (172).

Variations in OT activity at the OT receptor seem to follow a natural course, with a peak around early childhood (173). Differences are often seen when comparing groups of ages, but the OT activity cannot be used to categorically differ between young and old (174). Lack of OT activity is associated with aggression and other stress-related conditions (12) and oxytocin receptor activation is found to enhance the detection of stimuli that are paired with food reward and danger (175).

Diffuse spread of neuropeptides in the extracellular fluid following dendritic and focal release from axonal terminals has been suggested to contribute to the combined actions of neuromodulators and neurotransmitters (176). One of amygdala’s main functions is to regulate the relationship between emotions and motivation, e.g., scary and threatening stimuli on one side and positive feedback on the other side (177), and OT enhances amygdala-dependent, socially reinforced learning and emotional empathy in humans (178, 179).

There are reports that intranasal OT administration can favour social behaviour (180, 181) through influence on the amygdala (180). However, there are studies showing that low-dose OT supplements, in certain conditions, can also lead to aggressive behaviour (182, 183). Other studies on OT’s dysfunction suggest a possible link to the pathophysiological mechanisms behind schizophrenia and bipolar disorders (184), but also to anxiety (185). On the other hand, there are studies failing to confirm that OT may influence social behaviour and certain psychiatric conditions (186), and conclude instead that nasal administration has no desired behavioural effect at all (187, 188). Consequently, neither OT functions nor possible relations to social behaviour are clear (188).

Antibiotic cocktails that are not easily absorbed from the intestine in mice reduce oxytocin levels in the hypothalamus and were among the first to suggest that gut microbes can impact brain oxytocin. Administering antibiotics also disrupted social behaviour, which is not surprising when considering that oxytocin plays an essential role in social behaviour (189). This finding was consistent with studies in germ free mice that have abnormal social behaviour that can be normalised by colonisation with normal microbiota (190). Beyond its role in social behaviours, oxytocin has been gaining recognition for its ability to alter the immune response. The oxytocin receptor is found on both innate and adaptive leukocytes, and stimulation of the oxytocin receptor is largely thought to be anti-inflammatory (191). For example, oxytocin can reduce Toll-like receptor expression on neutrophils and can inhibit IL-6 production (192–194).

Research has described a link between OT, childhood trauma, and adolescent criminal activity (195). Less known is the relation between childhood trauma and aggression, but there is data from studies on individuals convicted of murder revealing a history of childhood trauma and lower levels of plasma OT compared to controls. Data from the same study also showed that the levels of plasma OT show an inverse correlation to childhood trauma in individuals convicted of homicide (196). Consideration of the current evidence led to the hypothesis suggesting that a possible defect in the oxytocinergic system could be responsible for the development of pathological aggression (196), and emphasising the role for OT in stress-related neuropsychiatric diseases (197, 198). An environment with chronic stress causes negative effects on our mental health and induces depression, anxiety, fatigue, and post-traumatic stress disorder (PTSD) (199). The prefrontal cortex is a key target region in stress-related neuropsychiatric disorders, and early life stress alters amygdala-prefrontal functional connectivity and sensitivity to the effects of OT treatment (200). OT is produced at various sites in the central nervous system (CNS) (198), and differences in the distribution of OTRs are strongly associated with the peptide’s physiological stress responses (197). In response to stress, OT regulates the HPA axis, and in particular, the feedback inhibition of corticosteroids (201–203). Studies have also revealed that patients with anxiety have significantly lower levels of plasma OT (203) and that patients with BPD have significantly reduced expression of OT receptors (OTRs); both patient groups compared to controls (197, 203). OT can amplify stress-induced fear responses involving the amygdala and thereby play a role in increasing fear and in the development of anxiety disorders such as PTSD, panic disorder, and phobias (197), and possibly a broader range of stress-related disorders (203). Of particular interest may be that serum OT levels during pregnancy have been associated with depressive symptoms in early pregnancy or postpartum and may serve as a predictive target for postpartum depression (204, 205).

Prenatal stress is also a vulnerability factor for development of anxiety and depression and it is mainly mediated by proinflammatory cytokines from the mother to the foetus. A recent study concluded that prenatal mood and anxiety disorders are linked to postpartum inflammation and women with prenatal psychiatric diagnosis assessed by the Structured Clinical Interview for the DSM-IV (SCID). These conditions also showed greater alterations of inflammatory markers, and the lowest levels of anti-inflammatory markers in pregnancy were associated with prenatal SCID mood/anxiety diagnoses (206).

On a molecular basis, T regulatory cell (Treg) variations seem involved with symptoms of anxiety. The FoxP3+ protein is a transcription factor, known for its controlling of gene activity, is also crucial for the normal production and function of Treg cells. These latter cells have a major role in the prevention of autoimmunity. One study found inverse associations between symptoms of anxiety and levels of FoxP3+ Tregs. Psychological distress was associated with other Treg subpopulations such as Helios+, Tim3+, and PD-1+. These results point at immunological tolerance mainly regulated by T cells, as a possible mechanism of prenatal psychological stress (207).

Results from an animal study underline that a consequence of being exposed to adverse experiences during gestation could be long-lasting changes potentially influencing the inflammatory response not only in the brain, but also in the periphery (208). Likewise, there are indications that prenatal negative events may influence the metabolism in organs such as the liver, in turn, causing vulnerability for developing metabolic related disorders (209).

Plasma levels of OT have been measured in a group of convicted violent criminals and compared to controls. Sixty percent of OT was naturally bound to IgG and transported in human plasma, and could thus, in turn modulate OTR signalling at more distant sites IgG from violently aggressive inmates was characterised by lower affinity for OT compared to controls, leading to decreased OT carrier capacity and reduced activation of the OTR in these subjects (12). In addition, animal studies have described that peripheral administration of OT together with human OT-reactive IgG administered to resident mice in a resident-intruder test, caused reduced c-fos activation in several involved regions of the brain regulating aggressive and defensive behaviour, a finding correlating with duration and the number of attacks (12). The data also establishes IgG as a carrier protein and that the IgG/OT complex (IOC) activates OTR, although with different kinetic abilities compared to OT alone. Our interpretation is that IgG has a modulating role in the effect of OT, supporting the importance of autoAbs and the IOC on the regulation of human behaviour. Aggressive behaviour was measured using the revised Buss-Perry aggression questionnaire by Bryant and Smith (12, 210). An additional observation was that the lack of effect of the IOC was linked to hostility subscale (12).

Although it cannot be generalised, OT concentrations obtained in specific settings are found to correlate positively with the severity of major depressive disorder (MDD), suggesting an association between the OT system and social behaviour in depressed patients (211). However, a negative correlation between symptom severity in depression, anxiety, and OT has also been reported (212). In other studies, OT has been characterised as a stress hormone, and there are reports linking elevated OT concentrations in human plasma to psychosocial stress in situations with the presence of unknown persons (183, 213). Levels of OT and vasopressin (VP) autoAbs are found to be low in moderate depression, and the respective levels of autoAbs correlate with scale scores for the disorder. However, levels of VP autoantibody correlate with plasma cortisol, and a blunted response to plasma cortisol in moderate depression during physical exercise supports the relevance of OT and VP autoantibodies for activity in the HPA stress axis and the symptoms of depression (90). Behavioural despair seems to promote the synthesis and secretion of OT in the brain and periphery, whereas specific brain-derived OT is important for depressive thinking (214).

Some clinical studies describe that plasma OT concentrations in depressed patients are higher than in healthy controls (215), but there are other studies claiming the opposite (216).

In a new study, a different model suggests an entirely new emotional disorder with OT deficiency as the main feature (217). Patients with vasopressin (VP) deficiency also displayed a deficiency of pituitary secreted OT, and interestingly, neuropsychological test results from the patients indicate both increased anxiety and a reduction in prosocial behaviour as major symptoms. Whether the suggestion of a new disorder will survive depends on future confirming and otherwise supporting studies. Moreover, further studies on the endocrine and immune processes behind neurotransmission within cortical and limbic brain circuits are needed (218) to fully comprehend depression as a disorder of possibly neuroimmune origin and perhaps also open up new diagnostic entities.

### 26RFa and aggression

The International Union of Basic and Clinical Pharmacology Committee on Receptor Nomenclature and Drug Classification (NC-IHUPAR), confirms the existence of the QRFP receptor/Arg-Phe-amide peptide 26RFa/glutamine RF peptide (26RFa). 26RFa, or QRFP, discovered in 2003 (219), is a neuropeptide consisting of 26 amino acids, and is mainly found in the hypothalamus. 26RFa is known for its regulation of hunger and glucose metabolism; however, much is unclear regarding its importance for emotional regulation. Studies of plasma levels in male perpetrators serving time for extremely violent crimes, compared to healthy non-violent controls, showed that the mean plasma levels of 26RFa were the same for the aggressors as for the controls, and that these plasma levels of 26RFa correlated positively with the HADS anxiety subscores in all the studied subjects and controls. It should be mentioned that in the study there were some issues with «outliers» in the group of violent perpetrators but not in the control group. After removing the high outliers, we saw positive correlations of 26RFa with HADS anxiety subscale and the subscale of hostility in the aggression scale for the inmates, and there was still a positive correlation between the plasma levels of 26RFa and anxiety in both groups studied. We found no correlations between 26RFa and other subscales of aggression or depression; however, an association between 26RFa and anxiety in human beings is kept open (12).

### Neuropeptide Y

Neuropeptide Y (NPY) is a 36-amino-acid neuropeptide involved in several different physiological processes in both the CNS and the peripheral nervous system. In the brain, it is produced at various sites, including the hypothalamus, and has several functions, such as causing increased food intake and fat storage, reducing anxiety and stress, reducing pain perception, lowering blood pressure, and controlling epileptic activity (220, 221). Studies on personality disorders with impulsivity and aggression showed that CSF NPY-like immunity (CSF NPY-LI) was directly correlated with measures of aggression and impulsivity and a composite measure of impulsive aggression (130).

Recent experiments on humans suggest that a variety of neuropeptide systems, including substance P (SP)/tachykinin, neuropeptide Y(NPY) and their G protein-coupled receptors, are involved in the regulation of mood disorders (222). Multiple sclerosis (MS) is commonly associated with depression (223), and alpha(α) calcitonin gene-related peptide (aCGRP), has a role together with neuropeptide Y (NPY), and substance P (SP); all three being potent immunomodulatory neuropeptides and possibly also as novel biomarkers in MS (224). Studies have shown that patients with depression have significantly lower levels of NPY in plasma compared to controls, but not in the CSF (130). In one study, a decrease in plasma NPY levels was observed, but without any significant difference between the levels seen in PTSD and depression. However, in patients where the stress was defined as chronic, there were statistically significantly higher plasma NPY levels compared to the control subjects with PTSD or depression. Although not clear, it is suggested that NPY has a role both in trauma and in depression (225).

Neuropeptide Y (NPY) is also the prototype of a phylogenetically well-conserved family of peptides and is a very potent orexigenic substance (226). Several peptide and hormone systems can affect NPY expression and release. NPY has potent antidepressant properties, and low central NPY plasma levels have been reported in major depression. Previous studies have shown that there are no significant differences in NPY IgG autoantibody (autoAb) affinities between patients with depression and controls (91). Future studies may find that quantitative changes in anti-NPY autoAbs plasma levels could be relevant to emotional changes and to patients with depression. Differences in affinity between NPY and IgG autoAbs may be linked to alterations in appetite and body weight (91).

## Discussion

The biological and pharmacological understanding of depression has developed notably since the early models of explanation, focusing on the presence of 5-hydroxyindoles in human CSF (227) and the differences between nor-imipramine and imipramine (228) to the conclusion that there is “no consistent evidence of the association” between depression and serotonin, perhaps with the exception that long-term use of antidepressants seems to lower the serotonin levels (229). Others (230), have argued that there is evidence for a low-grade inflammation being linked to depression, and refer to studies in which depressed patients and post mortem brains displayed greater amounts of cytokines (231–233). And there are also studies pointing out that acute tryptophan depletion and low plasma tryptophan in depression indicate 5-HT involvement (234). An hypotheses on the intersection of the monoaminergic and glutamatergic systems, and immune responses in the pathophysiology of depression have also been proposed (235).

**Figure 1:**
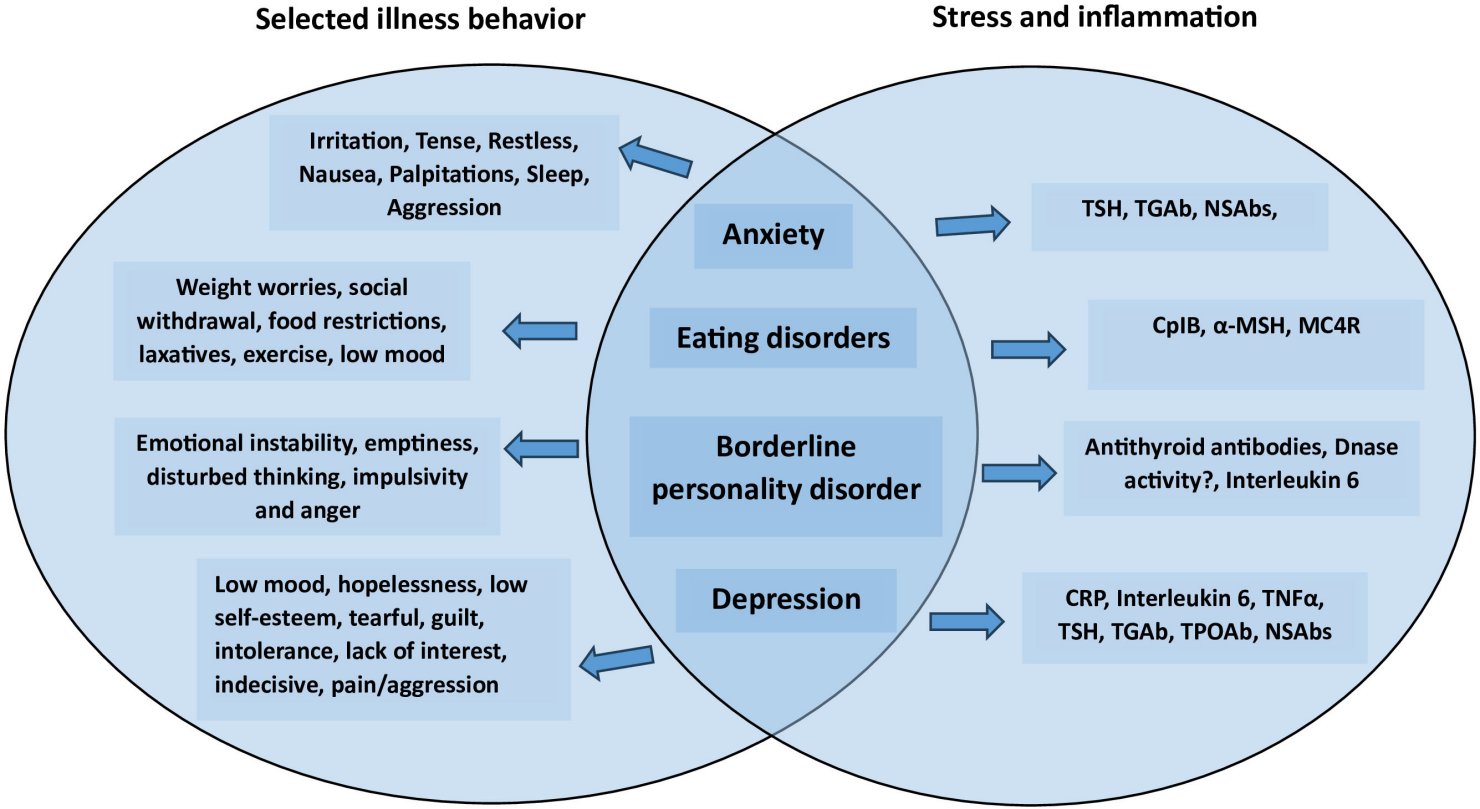
Psychiatric disorders featuring emotional problems with associated illness behavior and immunological molecular targets.

Undoubtedly, future approaches to depression and other emotional disorders will include psycho-immunological evaluations. As seen in **Figure 1**, common features for our alternative view on emotional disorders are stress and immunological reactions.

A study on psychiatric conditions associated with neural autoAbs over time showed that affective, cognitive, and psychotic symptoms remained relatively unchanged over the 1.5-years the study period lasted. Among the results reported, 63% of the patients were diagnosed with autoimmune psychiatric syndromes (236). Thus, psycho-immunology is gaining ground, and an important question has rightfully been raised (217) - whether we are moving towards new psychiatric entities? The developments in the studies on emotional disorders point in this direction.

**Figure 2:**
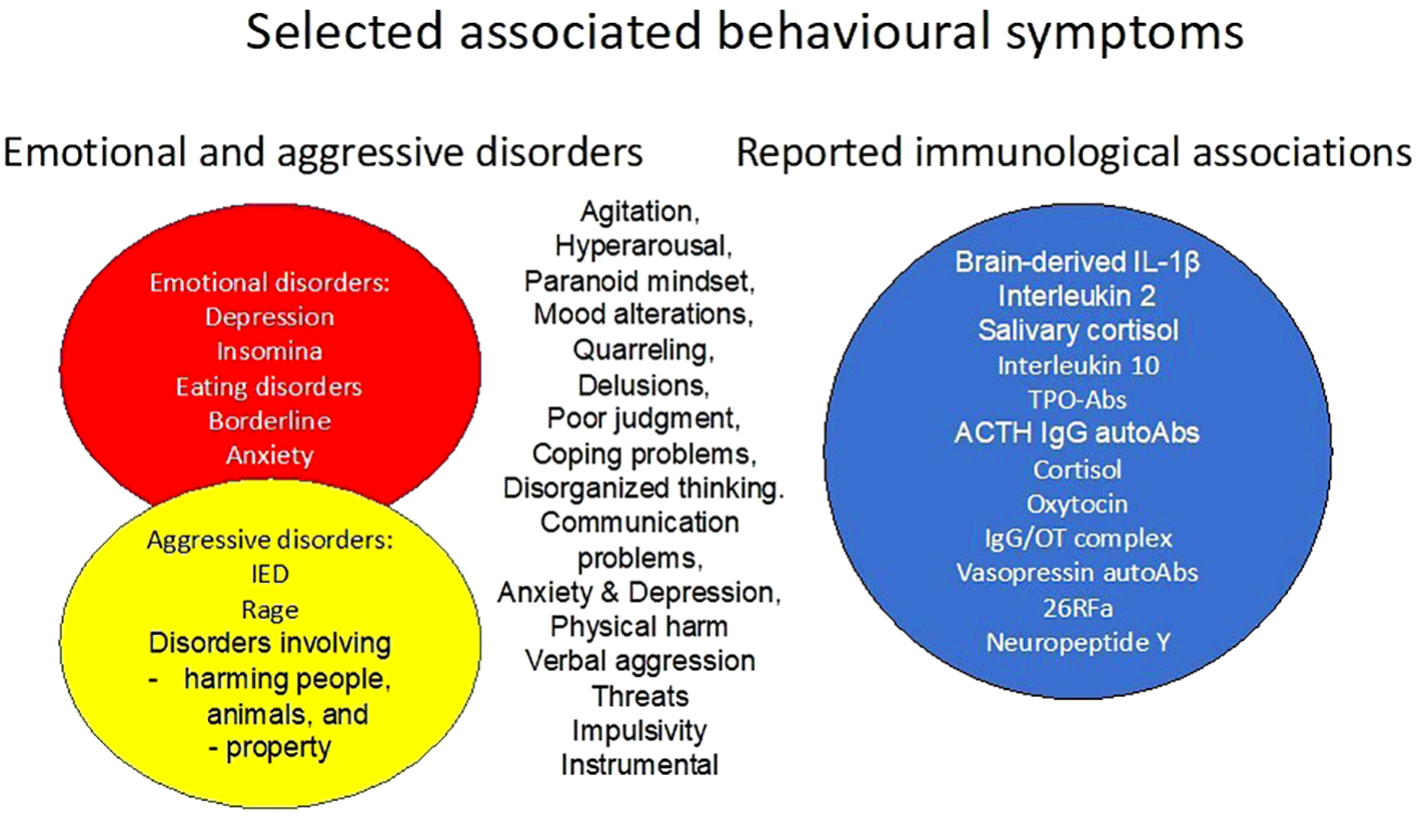
Selected associated behavioral symptoms of emotional problems and aggressive disorders, and their reported immunological associations.

There is also a hypothesis regarding the involvement of the gut microbiota, bacterial mimicry, and the possible effects of autoAbs on modulation and regulation of aggressive behaviour. Given the high overlap of behavioural symptoms and reported immunological associations (**Figure 2**) between aggressive and emotional disorders, there is a reason to hypothesise that there are convergent mechanisms involved. Modulation and regulation by IgG autoAbs represent a relatively new undercategory in psycho-immunology, or psycho-pharmaco-immunology, where studies are currently being conducted.

The existing diagnostic criteria for mental disorders are facing future challenges, and we believe that it is only a question of time before there will be enough evidence to include hard biological endpoints as part of new diagnostic criteria. However, the complexity of pathological human behaviour is immense, with all the possible concomitant disorders with overlapping symptoms, keeping up the level of challenges. Furthermore, the number of various levels of biological substrates involved is at present beyond our understanding.

More research will have a positive impact on global health issues, and there is much to gain regarding costs, not only personal but also for the society (**Table 1**).

**Table 1:** Psychiatric disorders featuring emotional problems and associated molecular targets.

Psychiatric disorders featuring emotional problems	Molecular targets	Selected references
Anxiety	TSH, TGAb, NSAbs,	(72, 73, 96)
Eating disorders	CpIB, α-MSH, MC4R	(5, 6, 9, 29)
Borderline personality disorder	Antithyroid antibodies, Dnase activity?, Interleukin 6	(33, 38, 42)
Depression	CRP, Interleukin 6, TNFα, TSH, TGAb, TPOAb, NSAbs	(83, 87, 96)

Senior psychiatrists and psychologists have been trained according to psychodynamic traditions, and today, new professionals in the field are receiving the same education even if knowledge about biomedical sciences is gradually incorporated into the respective educational programs. However, the behavioural issues we as professionals are faced with in our work with patients are the same as always; only the theoretical foundations are undergoing changes.

In this review, we have, on purpose, not included genetics in behavioural sciences and psychiatry, a field that we leave for others to review.

## References

[1] Sudo N Chida Y Aiba Y Sonoda J Oyama N Yu X-N. Postnatal microbial colonization programs the hypothalamic–pituitary–adrenal system for stress response in mice. J Physiol. (2004) 558:263–75. doi: 10.1113/jphysiol.2004.063388 PMC1664925 15133062

[2] Morris DJ Brem AS. Role of gut metabolism of adrenal corticosteroids and hypertension: clues gut-cleansing antibiotics give us. Physiol Genomics. (2019) 51:83–9. doi: 10.1152/physiolgenomics.00115.2018 30681907

[3] Morris DJ Ridlon JM. Glucocorticoids and gut bacteria: “The GALF Hypothesis” in the metagenomic era. Steroids. (2017) 125:1–13. doi: 10.1016/j.steroids.2017.06.002 28624548

[4] Fetissov SO. Neuropeptide-like signaling in the microbiota-gut-brain axis. Behav Brain Sci. (2019) 42:e70. doi: 10.1017/S0140525X18002765

[5] Fetissov SO Hökfelt T. On the origin of eating disorders: altered signaling between gut microbiota, adaptive immunity and the brain melanocortin system regulating feeding behavior. Curr Opin Pharmacol. (2019) 48:82–91. doi: 10.1016/j.coph.2019.07.004 31430598

[6] Fetissov SO Hallman J Oreland L Af Klinteberg B Grenbäck E Hulting A-L. Autoantibodies against α-MSH, ACTH, and LHRH in anorexia and bulimia nervosa patients. Proc Natl Acad Sci. (2002) 99:17155–60. doi: 10.1073/pnas.222658699 PMC139285 12486250

[7] Fetissov SO Hamze Sinno M Coëffier M Bole-Feysot C Ducrotté P Hökfelt T. Autoantibodies against appetite-regulating peptide hormones and neuropeptides: putative modulation by gut microflora. Nutrition. (2008) 24:348–59. doi: 10.1016/j.nut.2007.12.006 PMC7126273 18262391

[8] Sai A Shetty GB Shetty P N. HL. Influence of gut microbiota on autoimmunity: A narrative review. Brain Behav Immun Integr. (2024) 5:100046. doi: 10.1016/j.bbii.2024.100046

[9] Tennoune N Chan P Breton J Legrand R Chabane YN Akkermann K. Bacterial ClpB heat-shock protein, an antigen-mimetic of the anorexigenic peptide [alpha]-MSH, at the origin of eating disorders. Transl Psychiatry. (2014) 4:e458. 25290265 10.1038/tp.2014.98 PMC4350527

[10] Sadeghpour Heravi F. Gut microbiota and autoimmune diseases: mechanisms, treatment, challenges, and future recommendations. Curr Clin Microbiol Rep. (2024) 1181:18–33. doi: 10.1007/s40588-023-00213-6

[11] Værøy H Adori C Legrand R Lucas N Breton J Cottard C. Autoantibodies reactive to adrenocorticotropic hormone can alter cortisol secretion in both aggressive and nonaggressive humans. Proc Natl Acad Sci. (2018) 115:E6576–84. doi: 10.1073/pnas.1720008115 PMC6048475 29941562

[12] Værøy H Lahaye E Dubessy C Benard M Nicol M Cherifi Y. Immunoglobulin G is a natural oxytocin carrier which modulates oxytocin receptor signaling: relevance to aggressive behavior in humans. Discover Ment Health. (2023) 3:21. doi: 10.1007/s44192-023-00048-z PMC10587035 37983005

[13] Hutchison ED ed. Dimensions of human behavior: The changing life course. Emerita, Los Angeles: SAGE: Virginia Comonwealth University (2019).

[14] Mechanic D. Illness behaviour: an overview. In: Mchugh S Vallis TM, editors. Illness Behavior: A Multidisciplinary Model. Springer US, Boston, MA (1986).

[15] Monaghan LF. Illness and health behaviours. Key Concepts Med Sociology. (2022) 114.

[16] Oh H Marsiglia FF Pepin S Ayers S Wu S. Health behavior and attitudes during the COVID-19 Pandemic among vulnerable and underserved Latinx in the Southwest USA. Prev Sci. (2024) 25:279–90. doi: 10.1007/s11121-023-01512-6 PMC9978289 36862363

[17] Pires L Reis C Facão ARM Moniri A Marreiros A Drummond M. Fatigue and mental illness symptoms in long COVID: protocol for a prospective cohort multicenter observational study. JMIR Res Protoc. (2024) 13:e51820. doi: 10.2196/51820 38241071 PMC10837758

[18] Shiba K Cowden RG Counted V Vanderweele TJ Fancourt D. Associations of home confinement during COVID-19 lockdown with subsequent health and well-being among UK adults. Curr Psychol. (2024) 43:8532–41. doi: 10.1007/s12144-022-03001-5 PMC8922081 35309290

[19] Kessler RC Petukhova M Sampson NA Zaslavsky AM Wittchen HU. Twelve-month and lifetime prevalence and liftime morbid risk of anxiety and mood disorders in the United Stattes. Int J Methods Psychiatr Res. (2012) 21:169–84. doi: 10.1002/mpr.1359 PMC4005415 22865617

[20] Lahey B. Public Health significance of neuroticism. Am Psychol. (2009) 64:241–56. doi: 10.1037/a0015309 PMC2792076 19449983

[21] Mojtabai R Stuart EA Hwang I Susukida R Eaton WW Sampson N. Long-term effects of mental disoorders on employment in the National Comorbidity Survey ten-year follow up. Soc Psychiatry Psychiatr Epidemiol. (2015) 50:1657–68. doi: 10.1007/s00127-015-1097-z PMC4618045 26211661

[22] Wang PS Angermeyer M Borges G Bruffaerts R Chiu WT De Girolamo G. Delay and failure in treatment seekinng after first onset of mental disorders inn the World health Organization. World Psychiatry. (2007) 6:177–85. PMC2174579 18188443

[23] Bullis JR Boettcher H Sauer‐Zavala S Farchione TJ Barlow DH. What is an emotional ddisorder? Clin Psychol Sci Pract. (2019) 6. doi: 10.1111/cpsp.12278

[24] Himmerich H Bentley J Kan C Treasure J. Genetic risk factors for eating disorders: an update and insights into pathophysiology. Ther Adv Psychopharmacol. (2019) 9. doi: 10.1177/2045125318814734 PMC6378634 30800283

[25] Fan W Boston BA Kesterson RA Hruby VJ Cone RD. Role of melanocortinergic neurons in feeding and the agouti obesity syndrome. Nature. (1997) 385:165–8. doi: 10.1038/385165a0 8990120

[26] Morton G Cummings D Baskin D Barsh G Schwartz M. Central nervous system control of food intake and body weight. Nature. (2006) 443:289–95. doi: 10.1038/nature05026 16988703

[27] Jewett AI Shea JE. Folding on the chaperone: yield enhancement through loose binding. J Mol Biol. (2006) 363:945–57. doi: 10.1016/j.jmb.2006.08.040 16987526

[28] Mogk A Ruger-Herreros C Svoboda L Bukau B. Role of J-domain proteins in yeast physiology and protein quality control. J Mol Biol. (2024), 168484. 10.1016/j.jmb.2024.168484 38331212

[29] Lucas N Legrand R Bôle-Feysot C Breton J Coëffier M Akkermann K. Immunoglobulin G modulation of the melanocortin 4 receptor signaling in obesity and eating disorders. Trans Psychiatry. (2019) 9:87. doi: 10.1038/s41398-019-0422-9 PMC6372612 30755592

[30] Takagi K Legrand R Asakawa A Amitani H François M Tennoune N. Anti-ghrelin immunoglobulins modulate ghrelin stability and its orexigenic effect in obese mice and humans. Nat Commun. (2013) 4:2685. doi: 10.1038/ncomms3685 24158035 PMC3826639

[31] Seitz J Lahaye E Andreani NA Thomas B Takhlidjt S Chartrel N. Long-term dynamics of serum α-MSH and α-MSH-binding immunoglobulins with a link to gut microbiota composition in patients with anorexia nervosa. Neuroendocrinology. (2024). doi: 10.1159/000539316 PMC11460951 38852579

[32] Forte A Lessa PHC Chaves Filho AJM Aquino PEA Brito LM Pinheiro LC. Oxidative stress and inflammatory process in borderline personality disorder (BPD): a narrative review. Braz J Med Biol Res. (2023) 56:e12484. doi: 10.1590/1414-431x2023e12484 36946840 PMC10021502

[33] Geracioti TD Kling MA Post RM Gold PW. Antithyroid antibody-linked symptoms in borderline personality disorder. Endocr. (2003) 21:153–8. doi: 10.1385/ENDO:21:2 12897379

[34] Distel MAEA. Life events and borderline personality feaures. psychol Med. (2011) 41:849–60. doi: 10.1017/S0033291710001297 20594379

[35] Torgersen S Lygren S Øien PA Skre I Onstad S Edvardsen J. A twin study of personality disorders. Compr Psychiatry. (2000) 41:416–25. doi: 10.1053/comp.2000.16560 11086146

[36] Battle CL Shea MT Johnson DM Yen S Zlotnick C Zanarini MC. Childhood maltreatment associated with adult personality disorders: findings from the Collaborative Longitudinal Personality Disorders Study. J Pers Disord. (2004) 18:193–211. doi: 10.1521/pedi.18.2.193.32777 15176757

[37] Saccaro L Schilliger Z Dayer A Perroud N Piguet C. Inflammation, anxiety, and stress in bipolar disorder and borderline personality disorder: A narrative review. Neurosci Biobehav Rev. (2021) 127:184–92. doi: 10.1016/j.neubiorev.2021.04.017 33930472

[38] Ramesh R Sundaresh A Rajkumar RP Negi VS Vijayalakshmi MA Krishnamoorthy R. DNA hydolysing IgG catalytic antibodies. NPJ Schiophr. (2021) 7:13. doi: 10.1038/s41537-021-00143-6 PMC7910540 33637732

[39] Koenigsberg HW Yuan P Diaz GA Guerreri S Dorantes C Mayson S. Platelet protein kinase C and brain-derived neurotrophic factor levels in borderline personality disorder patients. Psychiatry Res. (2012) 199:92–7. doi: 10.1016/j.psychres.2012.04.026 PMC4128317 22633012

[40] Perroud N Salzmann A Prada P Nicastro R Hoeppli M-E Furrer S. Response to psychotherapy in borderline personality disorder and methylation status of the BDNF gene. Trans Psychiatry. (2013) 3:e207–7. doi: 10.1038/tp.2012.140 PMC3566720 23422958

[41] Kahl KG Rudolf S Stoeckelhuber BM Dibbelt L Gehl H-B Markhof K. Bone mineral density, markers of bone turnover, and cytokines in young women with borderline personality disorder with and without comorbid major depressive disorder. Am J Psychiatry. (2005) 162:168–74. doi: 10.1176/appi.ajp.162.1.168 15625216

[42] Kahl KG Bens S Ziegler K Rudolf S Dibbelt L Kordon A. Cortisol, the cortisol-dehydroepiandrosterone ratio, and pro-inflammatory cytokines in patients with current major depressive disorder comorbid with borderline personality disorder. Biol Psychiatry. (2006) 59:667–71. doi: 10.1016/j.biopsych.2005.08.001 16199015

[43] MacDowell KS Marsá MD Buenache E Villatoro JML Moreno B Leza JC. Inflammatory and antioxidant pathway dysfunction in borderline personality disorder. Psychiatry Res. (2020) 284:112782. doi: 10.1016/j.psychres.2020.112782 31955054

[44] Lopez-Villatoro JM Díaz-Marsá M De la Torre-Luque A MacDowell KS Prittwitz C Leza JC. Inflammatory and oxidative endophenotypes in borderline personality disorder. World J Psychiatry. (2023) 24:587–94. doi: 10.1080/15622975.2023.2183254 36919867

[45] Ruiz-Guerrero F del Barrio AG de la Torre-Luque A Ayad-Ahmed W Beato-Fernandez L Montes FP. Oxidative stress and inflammatory pathways in female eating disorders and borderline personality disorders with emotional dysregulation as linking factors with impulsivity and trauma. Psychoneuroendocrinology. (2023) 158:106383. doi: 10.1016/j.psyneuen.2023.106383 37714047

[46] Valizadeh P Momtazmanesh S Plazzi G Rezaei N. Connecting the dots: An updated review of the role of autoimmunity in narcolepsy and emerging immunotherapeutic approaches. Sleep Med. (2024) 113:378–96. doi: 10.1016/j.sleep.2023.12.005 38128432

[47] Kim EY Moudgil KD. Regulation of autoimmune inflammation by pro-inflammatory cytokines. Immunol Lett. (2008) 120:1–5. doi: 10.1016/j.imlet.2008.07.008 18694783 PMC2577081

[48] Kornum BR. Narcolepsy type 1: what have we learned from immunology? Sleep. (2020) 43:zsaa055. doi: 10.1093/sleep/zsaa055 32227223

[49] Kornum BR. Chapter 12 - Narcolepsy Type I as an autoimmune disorder. In: Swaab DF Buijs RM Lucassen PJ Salehi A Kreier F, editors. Handbook of Clinical Neurology. Elsevier (2021).

[50] Dietmann A Horn MP Schinkelshoek MS Fronczek R Salmen A Bargiotas P. Conventional autoantibodies against brain antigens are not routinely detectable in serum and CSF of narcolepsy type 1 and 2 patients. Sleep Med. (2020) 75:188–91. doi: 10.1016/j.sleep.2020.08.001 32858359

[51] Kornum BR Pizza F Knudsen S Plazzi G Jennum P Mignot E. Cerebrospinal fluid cytokine levels in type 1 narcolepsy patients very close to onset. Brain Behavior Immun. (2015) 49:54–8. doi: 10.1016/j.bbi.2015.03.004 PMC4567452 25771509

[52] Sheng D Li P Xiao Z Li X Liu J Xiao B. Identification of bidirectional causal links between gut microbiota and narcolepsy type 1 using Mendelian randomization. Sleep. (2024) 47:zsae004. doi: 10.1093/sleep/zsae004 38174762

[53] Chin W-C Huang Y-S Tang I Wang C-H. Long-term follow-up of symptom and quality of life changes in patients with narcolepsy during and after the COVID-19 pandemic. Sleep Biol Rhythms. (2024) 22:373–84. doi: 10.1007/s41105-024-00521-4 38962790 PMC11217227

[54] Prochazkova P Sonka K Roubalova R Jezkova J Nevsimalova S Buskova J. Investigation of anti-neuronal antibodies and disparity in central hypersomnias. Sleep Med. (2024) 113:220–31. doi: 10.1016/j.sleep.2023.11.039 38056084

[55] Moss RB Moll T El-Kalay M Kohne C Soo Hoo W Encinas J. Th1/Th2 cells in inflammatory disease states: therapeutic implications. Expert Opin Biol Ther. (2004) 4:1887–96. doi: 10.1517/14712598.4.12.1887 15571451

[56] Liblau RS Latorre D Kornum BR Dauvilliers Y Mignot EJ. The immunopathogenesis of narcolepsy type 1. Nat Rev Immunol. (2024) 24:33–48. doi: 10.1038/s41577-023-00902-9 37400646

[57] Lecendreux M Churlaud G Pitoiset F Regnault A Tran TA Liblau R. Narcolepsy type 1 is associated with a systemic increase and activation of regulatory T cells and with a systemic activation of global T cells. PloS One. (2017) 12:e0169836. doi: 10.1371/journal.pone.0169836 28107375 PMC5249232

[58] Deloumeau A Bayard S Coquerel Q Déchelotte P Bole-Feysot C. Increased immune complexes of hypocretin autoantibodies in narcolepsy. PLoS One (2010) 5:e13320. 20967199 10.1371/journal.pone.0013320 PMC2954157

[59] American Psychiatric Association. *Diagnostic and statistical manual of mental disorders*: DSM-5. 5th edn. Washington DC: American Psychiatric Association (2013).

[60] von Majewski K Kraus O Rhein C Lieb M Erim Y Rohleder N. Acute stress responses of autonomous nervous system, HPA axis, and inflammatory system in posttraumatic stress disorder. Trans Psychiatry. (2023) 13:36. doi: 10.1038/s41398-023-02331-7 PMC9894822 36732491

[61] Katrinli S Oliveira NCS Felger JC Michopoulos V Smith AK. The role of the immune system in posttraumatic stress disorder. Trans Psychiatry. (2022) 12:313. doi: 10.1038/s41398-022-02094-7 PMC9352784 35927237

[62] Wang Q Jia M Zhao Y Hui Y Pan J Yu H. Supplementation of sesamin alleviates stress-induced behavioral and psychological disorders via reshaping the gut microbiota structure. J Agric Food Chem. (2019) 67:12441–51. doi: 10.1021/acs.jafc.9b03652 31674783

[63] Dinse GE Parks CG Weinberg CR Co CA Wilkerson J Zeldin DC. Retracted: increasing prevalence of antinuclear antibodies in the United States. Arthritis Rheumatol. (2020) 72:1026–35. doi: 10.1002/art.41214 PMC7255943 32266792

[64] Dobbs K. Anxiety and Immunity: Affecting the body and mind. Thesis/Capstones/Creative Prrojects. (2022) 171.

[65] Ma Z Zhao M Zhao H Qu N. Causal role of immune cells in generalized anxiety disorder: Mendelian randomization study. Front Immunol. (2024) 14. doi: 10.3389/fimmu.2023.1338083 PMC10803460 38264647

[66] Zhang Q Zheng L Yang K. Assessment of bidirectional relationships between systemic lupus erythematosus and anxiety disorder: A two-sample Mendelian randomization study. Lupus. (2023) 32:471–6. doi: 10.1177/09612033231154784 36722420

[67] Moustafa AT Moazzami M Engel L Bangert E Hassanein M Marzouk S. Prevalence and metric of depression and anxiety in systemic lupus erythematosus: a systematic review and meta-analysis. Semin Arthritis rheumatism. (2020) 50:84–94. doi: 10.1016/j.semarthrit.2019.06.017 31303437

[68] Fernandez H Cevallos A Jimbo Sotomayor R Naranjo-Saltos F Mera Orces D Basantes E. Mental disorders in systemic lupus erythematosus: a cohort study. Rheumatol Int. (2019) 39:1689–95. doi: 10.1007/s00296-019-04423-4 31432225

[69] Wang J-L Ren C-H Feng J Ou C-H Liu L. Oleanolic acid inhibits mouse spinal cord injury through suppressing inflammation and apoptosis via the blockage of p38 and JNK MAPKs. Biomedicine Pharmacotherapy. (2020) 123:109752. doi: 10.1016/j.biopha.2019.109752 31924596

[70] Bale TL Contarino A Smith GW Chan R Gold LH Sawchenko PE. Mice deficient for corticotropin-releasing hormone receptor-2 display anxiety-like behaviour and are hypersensitive to stress. Nat Genet. (2000) 24:410–4. doi: 10.1038/74263 10742108

[71] Deng S Guo A Huang Z Guan K Zhu Y Chan C. The exploration of neuroinflammatory mechanism by which CRHR2 deficiency induced anxiety disorder. Prog Neuropsychopharmacol Biol Psychiatry. (2024) 128:110844. doi: 10.1016/j.pnpbp.2023.110844 37640149

[72] Yang L Wang X Kang H Gu B Ren Q Su D. Anti-Ro52 antibody is a risk factor for depression and anxiety in patients with connective tissue diseases. Res Square. (2023). doi: 10.21203/rs.3.rs-2904341/v1 38147314

[73] Wang X Wang Q Ren H Wang X Tang J Liao Y. The prevalence and clinical correlates of anxiety in Chinese patients with first-episode and drug-naive major depressive disorder at different ages of onset. J Affect Disord. (2023) 325:306–12. doi: 10.1016/j.jad.2023.01.032 36638965

[74] Howren MB Lamkin DM Suls J. Associations of depression with C-reactive protein, IL-1, and IL-6: a meta-analysis. Psychosomatic Med. (2009) 71:171–86. doi: 10.1097/PSY.0b013e3181907c1b 19188531

[75] Liu Y Ho RC-M Mak A. Interleukin (IL)-6, tumour necrosis factor alpha (TNF-α) and soluble interleukin-2 receptors (sIL-2R) are elevated in patients with major depressive disorder: a meta-analysis and meta-regression. J Affect Disord. (2012) 139:230–9. doi: 10.1016/j.jad.2011.08.003 21872339

[76] Valkanova V Ebmeier KP Allan CL. CRP, IL-6 and depression: a systematic review and meta-analysis of longitudinal studies. J Affect Disord. (2013) 150:736–44. doi: 10.1016/j.jad.2013.06.004 23870425

[77] Anderson RJ Freedland KE Clouse RE Lustman PJ. The prevalence of comorbid depression in adults with diabetes: a meta-analysis. Diabetes Care. (2001) 24:1069–78. doi: 10.2337/diacare.24.6.1069 11375373

[78] Boeschoten RE Braamse AM Beekman AT Cuijpers P van Oppen P Dekker J. Prevalence of depression and anxiety in multiple sclerosis: a systematic review and meta-analysis. J neurological Sci. (2017) 372:331–41. doi: 10.1016/j.jns.2016.11.067 28017241

[79] Isik A Koca SS Ozturk A Mermi O. Anxiety and depression in patients with rheumatoid arthritis. Clin Rheumatol. (2007) 26:872–8. doi: 10.1007/s10067-006-0407-y 16941197

[80] Shimo Y Cathomas F Lin H-Y Chan KL Parise LF Li L. Social stress induces autoimmune responses against the brain. Proc Natl Acad Sci. (2023) 120:e2305778120. doi: 10.1073/pnas.2305778120 38011565 PMC10710076

[81] Smith K de Torres I. A world of depression. Nature. (2014) 515:10–1038. doi: 10.1038/515180a

[82] Haapakoski R Mathieu J Ebmeier KP Alenius H Kivimäki M. Cumulative meta-analysis of interleukins 6 and 1β, tumour necrosis factor α and C-reactive protein in patients with major depressive disorder. Brain Behavior Immun. (2015) 49:206–15. doi: 10.1016/j.bbi.2015.06.001 PMC4566946 26065825

[83] Postal M Appenzeller S. The importance of cytokines and autoantibodies in depression. Autoimmun Rev. (2015) 14:30–5. doi: 10.1016/j.autrev.2014.09.001 25242344

[84] Miller AH Maletic V Raison CL. Inflammation and its discontents: the role of cytokines in the pathophysiology of major depression. Biol Psychiatry. (2009) 65:732–41. doi: 10.1016/j.biopsych.2008.11.029 PMC2680424 19150053

[85] Maes M Galecki P Chang YS Berk M. A review on the oxidative and nitrosative stress (O&NS) pathways in major depression and their possible contribution to the (neuro) degenerative processes in that illness. Prog Neuropsychopharmacol Biol Psychiatry. (2011) 35:676–92. doi: 10.1016/j.pnpbp.2010.05.004 20471444

[86] Moylan S Maes M Wray N Berk M. The neuroprogressive nature of major depressive disorder: pathways to disease evolution and resistance, and therapeutic implications. Mol Psychiatry. (2013) 18:595–606. doi: 10.1038/mp.2012.33 22525486

[87] Nuguru SP Rachakonda S Sripathi S Khan MI Patel N Meda RT. Hypothyroidism and depression. Cureus. (2022) 14. doi: 10.7759%2Fcureus.28201 10.7759/cureus.28416 PMC9509520 36171845

[88] Song Y Wang X Ma W Yang Y Yan S Sun J. Graves’ dissease as a driver of depression: a mechanistic insight. Front Endocrinol. (2023) 14. doi: 10.3389/fendo.2023.1162445 PMC10157224 37152963

[89] Zhang X Wang X Hu H Xu Y Zhang J Wang Z. Prevalence of self-reported thyroid disease among adults with depression. J Psychosomatic Res. (2024) 176:111557. doi: 10.1016/j.jpsychores.2023.111557 38056108

[90] Garcia FD Coquerel Q Kiive E Déchelotte P Harro J Fetissov SO. Autoantibodies reacting with vasopressin and oxytocin in relation to cortisol secretion in mild and moderate depression. Prog Neuropsychopharmacol Biol Psychiatry. (2011) 35:118–25. doi: 10.1016/j.pnpbp.2010.09.021 20932870

[91] Garcia FD Coquerel Q do Rego J-C Cravezic A Bole-Feysot C Kiive E. Anti-neuropeptide Y plasma immunoglobulins in relation to mood and appetite in depressive disorder. Psychoneuroendocrinology. (2012) 37:1457–67. doi: 10.1016/j.psyneuen.2012.01.015 22365482

[92] Iseme RA McEvoy M Kelly B Agnew L Attia J Walker FR. Autoantibodies and depression: evidence for a causal link? Neurosci Biobehav Rev. (2014) 40:62–79. doi: 10.1016/j.neubiorev.2014.01.008 24480318

[93] Raison CL Capuron L Miller AH. Cytokines sing the blues: inflammation and the pathogenesis of depression. Trends Immunol. (2006) 27:24–31. doi: 10.1016/j.it.2005.11.006 16316783 PMC3392963

[94] Schiepers OJG Wichers MC Maes M. Cytokines and major depression. Prog Neuropsychopharmacol Biol Psychiatry. (2005) 29:201–17. doi: 10.1016/j.pnpbp.2004.11.003 15694227

[95] Raison CL Miller AH. Is depression an inflammatory disorder? Curr Psychiatry Rep. (2011) 13:467–75. doi: 10.1007/s11920-011-0232-0 PMC3285451 21927805

[96] Zong S Correia-Hoffmann C Mané-Damas M Kappelmann N Molenaar PC van Grootheest G. Novel neuronal surface autoantibodies in plasma of patients with depression and anxiety. Trans Psychiatry. (2020) 10:404. doi: 10.1038/s41398-020-01083-y PMC7683539 33230123

[97] Ahlman H Nilsson O. The gut as the largest endocrine organ in the body. Ann Oncol. (2001) 12:S63–8. doi: 10.1093/annonc/12.suppl_2.S63 11762354

[98] Rehfeld JF. A centenary of gastrointestinal endocrinology. Hormone Metab Res. (2004) 36:735–41. doi: 10.1055/s-2004-826154 15655701

[99] Kulkarni S Ganz J Bayrer J Becker L Bogunovic M Rao M. Advances in enteric neurobiology: the “Brain” in the gut in health and disease. J Neurosci. (2018) 38:9346–54. doi: 10.1523/JNEUROSCI.1663-18.2018 PMC6209840 30381426

[100] Furness JB Stebbing MJ. The first brain: Species comparisons and evolutionary implications for the enteric and central nervous systems. Neurogastroenterol Motil. (2018) 30:e13234. doi: 10.1111/nmo.13234 29024273

[101] Mayer EA. Gut feelings: the emerging biology of gut–brain communication. Nat Rev Neurosci. (2011) 12:453–66. doi: 10.1038/nrn3071 PMC3845678 21750565

[102] Allen JM Mackos AR Jaggers RM Brewster PC Webb M Lin CH. Psychological stress disrupts intestinal epithelial cell function and mucosal integrity through microbe and host-directed processes. Gut Microbes. (2022) 14:2035661. doi: 10.1080/19490976.2022.2035661 35184677 PMC8865257

[103] Naseribafrouei A Hestad K Avershina E Sekelja M Linløkken A Wilson R. Correlation between the human fecal microbiota and depression. Neurogastroenterol Motil. (2014) 26:1155–62. doi: 10.1111/nmo.12378 24888394

[104] Jiang H Ling Z Zhang Y Mao H Ma Z Yin Y. Altered fecal microbiota composition in patients with major depressive disorder. Brain Behavior Immun. (2015) 48:186–94. doi: 10.1016/j.bbi.2015.03.016 25882912

[105] Kelly JR Borre Y O. B. C Patterson E El Aidy S Deane J. Transferring the blues: Depression-associated gut microbiota induces neurobehavioural changes in the rat. J Psychiatr Res. (2016) 82:109–18. doi: 10.1016/j.jpsychires.2016.07.019 27491067

[106] Konsman JP. Inflammation and depression: A nervous plea for psychiatry to not become immune to interpretation. Pharmaceuticals. (2019) 12. doi: 10.3390/ph12010029 PMC6469164 30769887

[107] Cryan JF Dinan TG. Mind-altering microorganisms: the impact of the gut microbiota on brain and behaviour. Nat Rev Neurosci. (2012) 13:701–12. doi: 10.1038/nrn3346 22968153

[108] Tillmann S Abildgaard A Winther G Wegener G. Altered fecal microbiota composition in the Flinders sensitive line rat model of depression. Psychopharmacology. (2019) 236:1445–57. doi: 10.1007/s00213-018-5094-2 PMC6599185 30470860

[109] Liu T Gu X Li L-X Li M Li B Cui X. Microbial and metabolomic profiles in correlation with depression and anxiety co-morbidities in diarrhoea-predominant IBS patients. BMC Microbiol. (2020) 20:168. doi: 10.1186/s12866-020-01841-4 32552668 PMC7302156

[110] McGaughey KD Yilmaz-Swenson T Elsayed NM Cruz DA Rodriguiz RM Kritzer MD. Relative abundance of Akkermansia spp. and other bacterial phylotypes correlates with anxiety- and depressive-like behavior following social defeat in mice. Sci Rep. (2019) 9:3281. doi: 10.1038/s41598-019-40140-5 30824791 PMC6397238

[111] McGuinness AJ Davis JA Dawson SL Loughman A Collier F O’hely M. A systematic review of gut microbiota composition in observational studies of major depressive disorder, bipolar disorder and schizophrenia. Mol Psychiatry. (2022) 27:1920–35. doi: 10.1038/s41380-022-01456-3 PMC9126816 35194166

[112] Xie T Fan X Pang H Zang T Wu N Liu J. Association between gut microbiota and its functional metabolites with prenatal depression in women. Neurobiol Stress. (2024) 28:100592. doi: 10.1016/j.ynstr.2023.100592 38075020 PMC10709355

[113] Caspani G Harvey M Swann J. Chapter 17 - metabolomics and the gut–brain axis. In: Hyland N Stanton C, editors. The Gut-Brain Axis, 2nd ed. Academic Press (2024).

[114] Cross T-WL Simpson AMR Lin C-Y Hottmann NM Bhatt AP Pellock SJ. Gut microbiome responds to alteration in female sex hormone status and exacerbates metabolic dysfunction. Gut Microbes. (2024) 16:2295429. doi: 10.1080/19490976.2023.2295429 38153260 PMC10761013

[115] Johnson D Thurairajasingam S Letchumanan V Chan K-G Lee L-H. Exploring the role and potential of probiotics in the field of mental health: major depressive disorder. Nutrients. (2021) 13:1728. doi: 10.3390/nu13051728 34065187 PMC8161395

[116] Chinna Meyyappan A Forth E Wallace CJK Milev R. Effect of fecal microbiota transplant on symptoms of psychiatric disorders: a systematic review. BMC Psychiatry. (2020) 20:299. doi: 10.1186/s12888-020-02654-5 32539741 PMC7294648

[117] D’Agata AL Wu J Welandawe MKV Dutra SVO Kane B Groer MW. Effects of early life NICU stress on the developing gut microbiome. Dev Psychobiology. (2019) 61:650–60. doi: 10.1002/dev.21826 PMC6588487 30697700

[118] Hantsoo L Kornfield S Anguera MC Epperson CN. Inflammation: A proposed intermediary between maternal stress and offspring neuropsychiatric risk. Biol Psychiatry. (2019) 85:97–106. doi: 10.1016/j.biopsych.2018.08.018 30314641 PMC6309506

[119] Marques TM Ganda-Mall JP Forsgård R Wall R Brummer RJ de Vos WM. Chapter 1 - correlating the gut microbiome to health and disease. In: Hyland N Stanton C, editors. The Gut-Brain Axis, 2nd ed. Academic Press (2024).

[120] Carlessi AS Borba LA Zugno AI Quevedo J Réus GZ. Gut microbiota-brain axis in depression: The role of neuroinflammation. Eurpean J Neurosci. (2021) 53:222–35. doi: 10.1111/ejn.14631 31785168

[121] Pesarico AP Vieira AT Rosa SG. Gut-microbiota-brain axis in depression: mechanisms and possible therapies. Front Behav Neurosci. (2023) 17. doi: 10.3389/fnbeh.2023.1221141 PMC10280164 37346896

[122] Petrovsky N. Towards a unified model of neuroendocrine–immune interaction. Immunol Cell Biol. (2001) 79:350–7. doi: 10.1046/j.1440-1711.2001.01029.x 11488982

[123] Kaelberer MM Buchanan KL Klein ME Barth BB Montoya MM Shen X. A gut-brain neural circuit for nutrient sensory transduction. Science. (2018) 361. doi: 10.1126/science.aat5236 PMC6417812 30237325

[124] Kaelberer MM Rupprecht LE Liu WW Weng P Bohórquez DV. Neuropod cells: the emerging biology of gut-brain sensory transduction. Annu Rev Neurosci. (2020) 43:337–53. doi: 10.1146/annurev-neuro-091619-022657 PMC7573801 32101483

[125] Schulte-Pelkum J Fritzler M Mahler M. Latest update on the Ro/SS-A autoantibody system. Autoimmun Rev. (2009) 8:632–7. doi: 10.1016/j.autrev.2009.02.010 19393201

[126] Chan EK. Anti-Ro52 autoantibody is common in systemic autoimmune rheumatic diseases and correlating with worse outcome when associated with interstitial lung disease in systemic sclerosis and autoimmune myositis. Clin Rev Allergy Immunol. (2022) 63:178–93. doi: 10.1007/s12016-021-08911-z 35040083

[127] Stanford MS Houston RJ Mathias CW Villemarette-Pittman NR Helfritz LE Conklin SM. Characterizing aggressive behavior. Assessment. (2003) 10:183–90. doi: 10.1177/1073191103010002009 12801190

[128] Weinschenker NJAS. Bimodal classification of aggression. Aggression Violent Behav. (2002) 7:237–50. doi: 10.1016/S1359-1789(01)00042-8

[129] Coccaro EF. Intermittent explosive disorder as a disorder of impulsive aggression for DSM-5. Am J Psychiatry. (2012) 169:577–88. doi: 10.1176/appi.ajp.2012.11081259 22535310

[130] Coccaro EF Lee R Liu T Mathé AA. Cerebrospinal fluid neuropeptide Y-like immunoreactivity correlates with impulsive aggression in human subjects. Biol Psychiatry. (2012) 72:997–1003. doi: 10.1016/j.biopsych.2012.07.029 22985695

[131] Meruelo AD Timmins MA Irwin MR Coccaro EF. Salivary cortisol awakening levelss are reduced in human subjects with intermittent explosive disorder compared to controls. Psychoneuroendocrinology. (2023) 151. doi: 10.1016/j.psyneuen.2023.106070 PMC10262314 36863129

[132] Kessler RC Coccaro EF Fava M Jaeger S Jin R Walters E. The prevalence and correlates of DSM-IV intermittent explosive disorder in the national comorbidity survey replication. Arch Gen Psychiatry. (2006) 63:669–78. doi: 10.1001/archpsyc.63.6.669 PMC1924721 16754840

[133] Modestino EJ Blum K Dennen CA Downs BW Bagchi D Llanos-Gomez L. Theorizing the role off dopamine polymorphic risk alleles with intermittent explosive disorder (IED), violent/aggressive behaavior aand addiction. J Pers Med. (2022) 12. doi: 10.3390/jpm12121946 PMC9784939 36556167

[134] Matthies S Rüsch N Weber M Lieb K Philipsen A Tuescher O. Small amygdala – high aggression? The role of the amygdala in modulating aggression in healthy subjects. World J Biol Psychiatry. (2012) 13:75–81. doi: 10.3109/15622975.2010.541282 22256828

[135] Haller J. The role of central and medial amygdala in normal and abnormal aggression: A review of classical approaches. Neurosci Biobehav Rev. (2018) 85:34–43. doi: 10.1016/j.neubiorev.2017.09.017 28918358

[136] Siegel A Schubert KL Shaikh MB. Neurotransmitters regulating defensive rage behavior in the cat. Neurosci Biobehav Rev. (1997) 21:733–42. doi: 10.1016/S0149-7634(96)00056-5 9415898

[137] de Boer SF Koolhaas JM. 5-HT1A and 5-HT1B receptor agonists and aggression: a pharmacological challenge of the serotonin deficiency hypothesis. Eur J Pharmacol. (2005) 526:125–39. doi: 10.1016/j.ejphar.2005.09.065 16310183

[138] Brown D Growdon J. L-Tryptophan administration potentiates serotonin-dependent myoclonic behavior in the rat. Neuropharmacology. (1980) 19:343–7. doi: 10.1016/0028-3908(80)90185-9 7383279

[139] Volpi-Abadie J Kaye AM Kaye AD. Serotonin syndrome. Ochsner J. (2013) 13:533–40. PMC3865832 24358002

[140] Marazziti D Rotondo A Presta S Pancioli-Guadagnucci ML Palego L Conti L. Role of serotonin in human aggressive behaviour. Aggressive Behav. (1993) 19:347–53. doi: 10.1002/(ISSN)1098-2337

[141] Duke AA Bègue L Bell R Eisen-Moul T. Revisiting the serotonin–aggression relation in humans: A meta-analysis. psychol Bull. (2013) 139:1148. doi: 10.1037/a0031544 23379963 PMC3718863

[142] Takahashi A Russo SJ. Link between the immune system and aggression. In: Martin CR Preedy VR Patel VB, editors. Handbook of Anger, Aggression, and Violence. Springer International Publishing, Cham (2023).

[143] Alperina E Idova G Zhukova E Zhanaeva S Kozhemyakina R. Cytokine variations within brain structures in rats selected for differences in aggression. Neurosci Lett. (2019) 692:193–8. doi: 10.1016/j.neulet.2018.11.012 30423398

[144] Siegel A Bhatt S Bhatt R Zalcman SS. The neurrobiologcal basis for development of Pham acological treatments of aggressive disorders. Curr Neuropharmaccology. (2007) 5:135–47. doi: 10.2174/157015907780866929 PMC2435345 18615178

[145] Zalcman SS Siegel A. The neurobiology of aggression and rage: role of cytokines. Brain Behav Immun. (2006) 20:507–14. doi: 10.1016/j.bbi.2006.05.002 16938427

[146] Alperina E Idova G Zhanaeva S. Rodent modeling of aggression: elucidating the role of cytokines in the brain. In: Martin CR Preedy VR Patel VB, editors. Handbook of Anger, Aggression, and Violence. Springer International Publishing, Cham (2023).

[147] Coccaro EF Lee R Breen EC Irwin MR. Plasma and cerebrospinal fluid inflammatory markers and human aggression. Neuropsychopharmacology. (2023) 48:1060–6. doi: 10.1038/s41386-023-01541-3 PMC10209212 36804488

[148] Majer AD Paitz RT Tricola GM Geduldig JE Litwa HP Farmer JL. The response to stressors in adulthood depends on the interaction between prenatal exposure to glucocorticoids and environmental context. Sci Rep. (2023) 13:6180. doi: 10.1038/s41598-023-33447-x 37061562 PMC10105737

[149] Kim Y-K Maes M. The role of the cytokine network in psychological stress. Acta Neuropsychiatrica. (2003) 15:148–55. doi: 10.1034/j.1601-5215.2003.00026.x 26983358

[150] Chong LS Rabkin AN Emhoff SM Barry-Menkhaus S Rivers AJ Lehrbach M. Childhood harsh parenting and later aggression: non-violent discipline and resting skin conductance as moderators. J Aggression Maltreatment Trauma. (2023) 32:537–54. doi: 10.1080/10926771.2022.2051658

[151] Barefoot JC. Developments in the measurement of hostility. In Friedman HS (Ed.), Hostility, coping, & health. American Psychological Association (1992), 13–31. doi: 10.1037/10105-001

[152] Haney TL Maynard KE Houseworth SJ Scherwitz LW. Interpersonal hostility assessment technique: description and validation against the criterion of coronary artery disease. J Pers Assess. (1996) 66:386–401. doi: 10.1207/s15327752jpa6602_16 8869579

[153] Song Q Lent M Murray-Close D Suo T Wang Q. Narrative processing and the forms and functions of aggressive behavior: Exploring the roles of physiological reactivity and gender. Narrative Inquiry. (2023). doi: 10.1075/ni

[154] Vaeroy H Schneider F Fetissov SO. Neurobiology of aggressive behavior—Role of autoantibodies reactive with stress-related peptide hormones. Front Psychiatry. (2019) 10. doi: 10.3389/fpsyt.2019.00872 PMC6904880 31866881

[155] Tennoune N Legrand R Ouelaa W Breton J Lucas N Bole-Feysot C. Sex-related effects of nutritional supplementation of Escherichia coli: Relevance to eating disorders. Nutrition. (2015) 31:498–507. doi: 10.1016/j.nut.2014.11.003 25701341

[156] Ren L Wang Y. Relationship between TPO-Ab with aggressive behavior in major mental disorders. Stress Brain. (2023) 3. doi: 10.26599/SAB.2023.9060006

[157] Coccaro EF Lee R Coussons-Read M. Elevated plasma inflammatory markers in individuals with intermittent explosive disorder and correlation with aggression in humans. JAMA Psychiatry. (2014) 71:158–65. doi: 10.1001/jamapsychiatry.2013.3297 24352431

[158] Trull TJ Jahng S Tomko RL Wood PK Sher KJ. Revised NESARC personality disorder diagnoses: gender, prevalence, and comorbidity with substance dependence disorders. J Pers Disord. (2010) 24:412–26. doi: 10.1521/pedi.2010.24.4.412 PMC3771514 20695803

[159] Lenzenweger MF Lane MC Loranger AW Kessler RC. DSM-IV personality disorders in the National Comorbidity Survey Replication. Biol Psychiatry. (2007) 62:553–64. doi: 10.1016/j.biopsych.2006.09.019 PMC2044500 17217923

[160] Azevedo J Vieira-Coelho M Castelo-Branco M Coelho R Figueiredo-Braga M. Impulsive and premeditated aggression in male offenders with antisocial personality disorder. PloS One. (2020) 15:e0229876. doi: 10.1371/journal.pone.0229876 32142531 PMC7059920

[161] Wang T-Y Lee S-Y Hu M-C Chen S-L Chang Y-H Chu C-H. More inflammation but less brain-derived neurotrophic factor in antisocial personality disorder. Psychoneuroendocrinology. (2017) 85:42–8. doi: 10.1016/j.psyneuen.2017.08.006 28810156

[162] Haller J Kruk MR. Normal and abnormal aggression: human disorders and novel laboratory models. Neurosci Biobehav Rev. (2006) 30:292–303. doi: 10.1016/j.neubiorev.2005.01.005 16483889

[163] Nelson RJ Trainor BC. Neural mechanisms of aggression. Nat Rev Neurosci. (2007) 8:536–46. doi: 10.1038/nrn2174 17585306

[164] de Boer SF Olivier B Veening J Koolhaas JM. The neurobiology of offensive aggression: Revealing a modular view. Physiol Behav. (2015) 146:111–27. doi: 10.1016/j.physbeh.2015.04.040 26066717

[165] Chrousos GP Gold PW. The concepts of stress and stress system disorders: overview of physical and behavioral homeostasis. JAMA. (1992) 267:1244–52. doi: 10.1001/jama.1992.03480090092034 1538563

[166] Gillespie CF Nemeroff CB. Hypercortisolemia and depression. Psychosomatic Med. (2005) 67:S26–8. doi: 10.1097/01.psy.0000163456.22154.d2 15953796

[167] Vedhara K Miles J Bennett P Plummer S Tallon D Brooks E. An investigation into the relationship between salivary cortisol, stress, anxiety and depression. Biol Psychol. (2003) 62:89–96. doi: 10.1016/S0301-0511(02)00128-X 12581685

[168] van Honk J Peper JS Schutter DJLG. Testosterone reduces unconscious fear but not consciously experienced anxiety: implications for the disorders of fear and anxiety. Biol Psychiatry. (2005) 58:218–25. doi: 10.1016/j.biopsych.2005.04.003 15939408

[169] Schaefer JM Fetissov SO Legrand R Claeyssens S Hoekstra PJ Verhulst FC. Corticotropin (ACTH)-reactive immunoglobulins in adolescents in relation to antisocial behavior and stress-induced cortisol response. The TRAILS study. Psychoneuroendocrinology. (2013) 38:3039–47. doi: 10.1016/j.psyneuen.2013.08.015 24103889

[170] Fetissov SO Hallman J Nilsson I Lefvert AK Oreland L Hökfelt T. Aggressive behavior linked to corticotropin-reactive autoantibodies. Biol Psychiatry. (2006) 60:799–802. doi: 10.1016/j.biopsych.2006.03.081 16876133

[171] Quintana DS Rokicki J van der Meer D Alnæs D Kaufmann T Córdova-Palomera A. Oxytocin pathway gene networks in the human brain. Nat Commun. (2019) 10:668. doi: 10.1038/s41467-019-08503-8 30737392 PMC6368605

[172] Zhan S Qi Z Cai F Gao Z Xie J Hu J. Oxytocin neurons mediate stress-inducedd social memory impairment. Curr Biol. (2023) 34:36–45. 10.1016/j.cub.2023.11.037 38103551

[173] Rokicki J Kaufmann T de Lange A-MG van der Meer D Bahrami S Sartorius AM. Oxytocin receptor expression patterns in the human brain across development. Neuropsychopharmacology. (2022) 47:1550–60. doi: 10.1038/s41386-022-01305-5 PMC9205980 35347267

[174] Audunsdottir K Quintana DS. Oxytocin’s dynamic role across the lifespan. Aging Brain. (2022) 2:100028. doi: 10.1016/j.nbas.2021.100028 36908876 PMC9997153

[175] Fam J Holmes N Westbrook RF. Stimulating oxytocin receptors in the basolateral amygdala enhances stimulus processing: Differential and consistent effects for stimuli paired with fear versus sucrose in extinction and reversal learning. Psychoneuroendocrinology. (2024) 160:106917. doi: 10.1016/j.psyneuen.2023.106917 38071877

[176] Landgraf R Neumann ID. Vasopressin and oxytocin release within the brain: a dynamic concept of multiple and variable modes of neuropeptide communication. Front Neuroendocrinol. (2004) 25:150–76. doi: 10.1016/j.yfrne.2004.05.001 15589267

[177] Janak PH Tye KM. From circuits to behaviour in the amygdala. Nature. (2015) 517:284–92. doi: 10.1038/nature14188 PMC4565157 25592533

[178] Hurlemann R Patin A Onur OA Cohen MX Baumgartner T Metzler S. Oxytcin enhances amygdala-dependant, socially reinforced learning and emotional empathy in humans. J Neurosci. (2010) 30:4999–5007. doi: 10.1523/JNEUROSCI.5538-09.2010 20371820 PMC6632777

[179] Huber D Veinante P Stoop R. Vasopressin and oxytocin excite distinct neuronal populations in the central amygdala. Science. (2005) 308:245–8. doi: 10.1126/science.1105636 15821089

[180] Quintana DS Westlye LT Alnæs D Rustan Ø.G Kaufmann T Smerud KT. Low dose intranasal oxytocin delivered with Breath Powered device dampens amygdala response to emotional stimuli: A peripheral effect-controlled within-subjects randomized dose-response fMRI trial. Psychoneuroendocrinology. (2016) 69:180–8. doi: 10.1016/j.psyneuen.2016.04.010 27107209

[181] Quintana DS Westlye LT Rustan Ø.G Tesli N Poppy CL Smevik H. Low-dose oxytocin delivered intranasally with Breath Powered device affects social-cognitive behavior: a randomized four-way crossover trial with nasal cavity dimension assessment. Trans Psychiatry. (2015) 5:e602–2. doi: 10.1038/tp.2015.93 PMC5068727 26171983

[182] Ne'Eman R Perach-Barzilay N Fischer-Shofty M Atias A Shamay-Tsoory SG. Intranasal administration of oxytocin increases human aggressive behavior. Hormones Behav. (2016) 80:125–31. doi: 10.1016/j.yhbeh.2016.01.015 26862988

[183] Neumann ID Slattery DA. Oxytocin in general anxiety and social fear: a translational approach. Biol Psychiatry. (2016) 79:213–21. doi: 10.1016/j.biopsych.2015.06.004 26208744

[184] Quintana DS Dieset I Elvsåshagen T Westlye LT Andreassen OA. Oxytocin system dysfunction as a common mechanism underlying metabolic syndrome and psychiatric symptoms in schizophrenia and bipolar disorders. Front Neuroendocrinol. (2017) 45:1–10. doi: 10.1016/j.yfrne.2016.12.004 28049009

[185] Quintana DS Outhred T Westlye LT Malhi GS Andreassen OA. The impact of oxytocin administration on brain activity: a systematic review and meta-analysis protocol. Systematic Rev. (2016) 5:205. doi: 10.1186/s13643-016-0386-2 PMC5129649 27899138

[186] Alvares GA Quintana DS Whitehouse AJO. Beyond the hype and hope: Critical considerations for intranasal oxytocin research in autism spectrum disorder. Autism Res. (2017) 10:25–41. doi: 10.1002/aur.1692 27651096

[187] Dadds MR Macdonald E Cauchi A Williams K Levy F Brennan J. Nasal oxytocin for social deficits in childhood autism: A randomized controlled trial. J Autism Dev Disord. (2014) 44:521–31. doi: 10.1007/s10803-013-1899-3 23888359

[188] Quintana DS Guastella AJ. An allostatic theory of oxytocin. Trends Cogn Sci. (2020) 24:515–28. doi: 10.1016/j.tics.2020.03.008 32360118

[189] Desbonnet L Clarke G Traplin A O’sullivan O Crispie F Moloney RD. Gut microbiota depletion from early adolescence in mice: Implications for brain and behaviour. Brain Behavior Immun. (2015) 48:165–73. doi: 10.1016/j.bbi.2015.04.004 25866195

[190] Desbonnet L Clarke G Shanahan F Dinan TG Cryan JF. Microbiota is essential for social development in the mouse. Mol Psychiatry. (2014) 19:146–8. doi: 10.1038/mp.2013.65 PMC3903109 23689536

[191] Mehdi SF Pusapati S Khenhrani RR Farooqi MS Sarwar S Alnasarat A. Oxytocin and related peptide hormones: candidate anti-inflammatory therapy in early stages of sepsis. Front Immunol. (2022) 13. doi: 10.3389/fimmu.2022.864007 PMC9102389 35572539

[192] Işeri SÖ Şener G Sağlam B Gedik N Ercan F Yeğen BÇ. Oxytocin ameliorates oxidative colonic inflammation by a neutrophil-dependent mechanism. Peptides. (2005) 26:483–91. doi: 10.1016/j.peptides.2004.10.005 15652655

[193] Işeri SÖ Şener G Sağlam B Gedik N Ercan F Yeğen BÇ. Oxytocin protects against sepsis-induced multiple organ damage: role of neutrophils. J Surg Res. (2005) 126:73–81. doi: 10.1016/j.jss.2005.01.021 15916978

[194] Szeto A Nation DA Mendez AJ Dominguez-Bendala J Brooks LG Schneiderman N. Oxytocin attenuates NADPH-dependent superoxide activity and IL-6 secretion in macrophages and vascular cells. Am J Physiology-Endocrinology Metab. (2008) 295:E1495–501. doi: 10.1152/ajpendo.90718.2008 PMC2603556 18940936

[195] Hesselink A. Adverse childhood experiences (ACEs) and the link to antisocial, delinquent, and criminal behaviors. In Criminal Behavior-The Underlyings, and Contemporary Applications. IntechOpen (2023).

[196] Goh KK Lu M-L Jou S. Childhood trauma and aggression in persons convicted for homicide: an exploratory study examines the role of plasma oxytocin. Front Psychiatry. (2021) 12. doi: 10.3389/fpsyt.2021.719282 PMC8415833 34484006

[197] Zhang S Zhang Y-D Shi D-D Wang Z. Therapeutic uses of oxytocin in stress-related neuropsychiatric disorders. Cell Bioscience. (2023) 13:216. doi: 10.1186/s13578-023-01173-6 38017588 PMC10683256

[198] Shi D-D Zhang Y-D Ren Y-Y Peng S-Y Yuan T-F Wang Z. Predictable maternal separation confers adult stress resilience via the medial prefrontal cortex oxytocin signaling pathway in rats. Mol Psychiatry. (2021) 26:7296–307. doi: 10.1038/s41380-021-01293-w 34561611

[199] Santomauro DF Mantilla Herrera AM Shadid J Zheng P Ashbaugh C Pigott DM. Global prevalence and burden of depressive and anxiety disorders in 204 countries and territories in 2020 due to the COVID-19 pandemic. Lancet. (2021) 398:1700–12. doi: 10.1016/S0140-6736(21)02143-7 PMC8500697 34634250

[200] Fan Y Herrera-Melendez AL Pestke K Feeser M Aust S Otte C. Early life stress modulates amygdala-prefrontal functional connectivity: Implications for oxytocin effects. Hum Brain Mapp. (2014) 35:5328–39. doi: 10.1002/hbm.22553 PMC6869775 24862297

[201] Neumann ID Wigger A Torner Holsboer Landgraf. Brain oxytocin inhibits basal and stress-induced activity of the hypothalamo-pituitary-adrenal axis in male and female rats: partial action within the paraventricular nucleus. J Neuroendocrinol. (2000) 12:235–43. doi: 10.1046/j.1365-2826.2000.00442.x 10718919

[202] Swaab DF Bao A-M Lucassen PJ. The stress system in the human brain in depression and neurodegeneration. Ageing Res Rev. (2005) 4:141–94. doi: 10.1016/j.arr.2005.03.003 15996533

[203] Diaz-Marsá M López-Villatoro JM de la Torre-Luque A Macdowell KS Galvez-Merlin A Gómez del Barrio A. Decreased oxytocin plasma levels and oxytocin receptor expression associated with aggressive behavior in aggressive-impulsive disorders. J Psychiatr Res. (2023) 170:200–6. doi: 10.1016/j.jpsychires.2023.12.032 38157667

[204] Skrundz M Bolten M Nast I Hellhammer DH Meinlschmidt G. Plasma oxytocin concentration during pregnancy is associated with development of postpartum depression. Neuropsychopharmacology. (2011) 36:1886–93. doi: 10.1038/npp.2011.74 PMC3154107 21562482

[205] Ma X Wei Q Jiang Z Shi Y Zhang Y Shi H. The role of serum oxytocin levels in the second trimester in regulating prenatal anxiety and depression: A sample from Shanghai Maternal-Child Pairs Cohort study. J Affect Disord. (2020) 264:150–6. doi: 10.1016/j.jad.2019.12.019 32056744

[206] Mancuso RA Ross KM Accortt E Coussons-Read M Okun ML Irwin J. Prenatal mood and anxiety disorders and associated cytokine changes. J Affect Disord. (2024) 347:635–44. doi: 10.1016/j.jad.2023.12.014 PMC11375962 38070749

[207] Wiley KS Kwon D Knorr DA Fox MM. Regulatory T-cell phenotypes in prenatal psychological distress. Brain Behavior Immun. (2024) 116:62–9. doi: 10.1016/j.bbi.2023.11.033 PMC11402516 38016492

[208] Petrillo G D’Aprile I Mazzelli M Riva MA Cattaneo A. Exposure to prenatal stress and the development of a vulnerable or resilient phenotype at adulthood: potential role of microglia activation. Psychoneuroendocrinology. (2024) 160:106925.

[209] D’aprile I Petrillo G Mazzelli M Zonca V Riva MA Cattaneo A. -Long-term effect of prenatal stress exposure on metabolism in vulnerable and resilient animals. Psychoneuroendocrinology. (2024) 160:106885. doi: 10.1016/j.psyneuen.2023.106885

[210] Bryant F Smith BD. Refining the architecture of aggression: a measurement model for the Buss-Perry. J Res Pers. (2001) 35:138–67. doi: 10.1006/jrpe.2000.2302

[211] Cyranowski JM Hofkens TL Frank E Seltman H Cai H-M Amico JA. Evidence of dysregulated peripheral oxytocin release among depressed women. Psychosomatic Med. (2008) 70:967. doi: 10.1097/PSY.0b013e318188ade4 PMC3397424 19005082

[212] Scantamburlo G Hansenne M Fuchs S Pitchot W Marechal P Pequeux C. Plasma oxytocin levels and anxiety in patients with major depression. Psychoneuroendocrinology. (2007) 32:407–10. doi: 10.1016/j.psyneuen.2007.01.009 17383107

[213] Tabak BA Leng G Szeto A Parker KJ Verbalis JG Ziegler TE. Advances in human oxytocin measurement: challenges and proposed solutions. Mol Psychiatry. (2023) 28:127–40. doi: 10.1038/s41380-022-01719-z PMC9812775 35999276

[214] Yan Y Wang Y-L Su Z Zhang Y Guo S-X Liu A-J. Effect of oxytocin on the behavioral activity in the behavioral despair depression rat model. Neuropeptides. (2014) 48:83–9. doi: 10.1016/j.npep.2014.01.001 24444823

[215] Holt-Lunstad J Birmingham W Light KC. The influence of depressive symptomatology and perceived stress on plasma and salivary oxytocin before, during and after a support enhancement intervention. Psychoneuroendocrinology. (2011) 36:1249–56. doi: 10.1016/j.psyneuen.2011.03.007 21507578

[216] Engel S Laufer S Knaevelsrud C Schumacher S. The endogenous oxytocin system in depressive disorders: A systematic review and meta-analysis. Psychoneuroendocrinology. (2019) 101:138–49. doi: 10.1016/j.psyneuen.2018.11.011 30458371

[217] Verbalis JG. Oxytocin deficiency — a ‘new’ human disorder? Nat Rev Endocrinol. (2023) 19:505–6. 10.1038/s41574-023-00870-z 37430034

[218] McGuinness B Harkin A. Rodent models of stress-induced depression: the link between stress and immune system related changes. In: Müller N Myint A-M Schwarz MJ, editors. Immunology and Psychiatry: From Basic Research to Therapeutic Interventions. Springer International Publishing, Cham (2015).

[219] Chartrel N Dujardin C Anouar Y Leprince J Decker A Clerens S. Identification of 26RFa, a hypothalamic neuropeptide of the RFamide peptide family with orexigenic activity. PNAS. (2003) 100:15247–52. doi: 10.1073/pnas.2434676100 PMC299975 14657341

[220] Tatemoto K. Neuropeptide Y and related peptides. Springer (2004).

[221] Colmers WF El Bahh B. Neuropeptide Y and epilepsy. Epilepsy Currents. (2003) 3:53–8. doi: 10.1111/j.1535-7597.2003.03208.x PMC321170 15309085

[222] Swapnali B Julio A Wen Z Anna S Irene M Josee PH. Expression of substance P, NPY and their Receptors Is Altered in Major Depression. bioRxiv. (2022). doi: 10.1101/2022.12.14.516867

[223] Siddiqi SH Kletenik I Anderson MC Cavallari M Chitnis T Glanz BI. Lesion network localization of depression in multiple sclerosis. Nat Ment Health. (2023) 1:36–44. doi: 10.1038/s44220-022-00002-y PMC13316769 42383171

[224] Al-Keilani M Almomani BA Jaradat SA Al-Sawalha NA Qawasmeh MA. Alpha calcitonin gene related peptide, neuropeptide Y, and substance P as biomarkers for diagnosis and disease activity and severity in MS. CNS Neurological Disord. (2023) 23:512–24. doi: 10.2174/1871527322666230403130540 37013432

[225] Tural UAI. Neuropeptide Y i PTSD, MDD and chronic stress. J Neurosci Res. (2020) 5:950–63. doi: 10.1002/jnr.24589 32048334

[226] Hökfelt T Stanic D Sanford SD Gatlin JC Nilsson I Paratcha G. NPY and its involvement in axon guidance, neurogenesis, and feeding. Nutrition. (2008) 24:860–8. doi: 10.1016/j.nut.2008.06.010 18725084

[227] Ashcroft G Sharman D. 5-Hydroxyindoles in human cerebrospinal fluids. Nature. (1960) 186:1050–1. doi: 10.1038/1861050a0 13794708

[228] Abood L Kimizuka H Rogeness G Biel J. Some antidepressant drugs and their mechanism of action on excitable membranes. Ann New York Acad Sci. (1963) 107:1139–46. doi: 10.1111/j.1749-6632.1963.tb13356.x 14010697

[229] Moncrieff J Cooper RE Stockmann T Amendola S Hengartner MP Horowitz MA. The serotonin theory of depression: a systematic umbrella review of the evidence. Mol Psychiatry. (2023) 28:3243–56. doi: 10.1038/s41380-022-01661-0 PMC10618090 35854107

[230] Pastis I Santos MG Paruchuri A. Exploring the role of inflammation in major depressive disorder: beyond the monoamine hypothesis. Front Behav Neurosci. (2024) 17:1282242. doi: 10.3389/fnbeh.2023.1282242 38299049 PMC10829100

[231] Hajebrahimi B Bagheri M Hassanshahi G Nazari M Bidaki R Khodadadi H. The adapter proteins of TLRs, TRIF and MYD88, are upregulated in depressed individuals. Int J Psychiatry Clin Pract. (2014) 18:41–4. doi: 10.3109/13651501.2013.859708 24168294

[232] Figueroa-Hall LK Paulus MP Savitz J. Toll-like receptor signaling in depression. Psychoneuroendocrinology. (2020) 121:104843. doi: 10.1016/j.psyneuen.2020.104843 32911436 PMC7883590

[233] Enache D Pariante CM Mondelli V. Markers of central inflammation in major depressive disorder: a systematic review and meta-analysis of studies examining cerebrospinal fluid, positron emission tomography and post-mortem brain tissue. Brain behavior Immun. (2019) 81:24–40. doi: 10.1016/j.bbi.2019.06.015 31195092

[234] Jauhar S Arnone D Baldwin DS Bloomfield M Browning M Cleare AJ. A leaky umbrella has little value: evidence clearly indicates the serotonin system is implicated in depression. Mol Psychiatry. (2023) 28:3149–52. doi: 10.1038/s41380-023-02095-y PMC10618084 37322065

[235] Kadriu B Farmer CA Yuan P Park LT Deng Z-D Moaddel R. The kynurenine pathway and bipolar disorder: intersection of the monoaminergic and glutamatergic systems and immune response. Mol Psychiatry. (2021) 26:4085–95. doi: 10.1038/s41380-019-0589-8 PMC7225078 31732715

[236] Hansen N Rentzsch K Hirschel S Bartels C Wiltfang J Malchow B. Long-term course of neural autoantibody-associated psychiatric disorders: retrospective data from a specifically immunopsychiatric outpatient clinic. Antibodies. (2023) 12:34. doi: 10.3390/antib12020034 37218900 PMC10204564

